# Spice-Derived Bioactive Ingredients: Potential Agents or Food Adjuvant in the Management of Diabetes Mellitus

**DOI:** 10.3389/fphar.2018.00893

**Published:** 2018-08-22

**Authors:** Aminu Mohammed, Md. Shahidul Islam

**Affiliations:** ^1^Department of Biochemistry, Faculty of Life Sciences, Ahmadu Bello University, Zaria, Nigeria; ^2^Department of Biochemistry, School of Life Sciences, University of KwaZulu-Natal, Durban, South Africa

**Keywords:** adjuvant, diabetes mellitus, hypoglycemic, *in vitro*, *in vivo*, spices

## Abstract

Spices possess tremendous therapeutic potential including hypoglycemic action, attributed to their bioactive ingredients. However, there is no study that critically reviewed the hypoglycemic potency, safety and the bioavailability of the spice-derived bioactive ingredients (SDBI). Therefore, the aim of the study was to comprehensively review all published studies regarding the hypoglycemic action of SDBI with the purpose to assess whether the ingredients are potential hypoglycemic agents or adjuvant. Factors considered were concentration/dosages used, the extent of blood glucose reduction, the IC_50_ values, and the safety concern of the SDBI. From the results, cinnamaldehyde, curcumin, diosgenin, thymoquinone (TQ), and trigonelline were showed the most promising effects and hold future potential as hypoglycemic agents. Conclusively, future studies should focus on improving the tissue and cellular bioavailability of the promising SDBI to achieve greater potency. Additionally, clinical trials and toxicity studies are with these SDBI are warranted.

## Introduction

Diabetes mellitus (DM) is a chronic metabolic disorder characterized by hyperglycemia resulting from the malfunction in insulin secretion and/or insulin action, both leading to impair metabolism of carbohydrates, lipids, and proteins (ADA, 2015). The prevalence of DM is increasing exponentially to over 425 million people globally, and this figure is likely to rise to 629 million by 2045 (IDF, 2017; Ogurtsova et al., 2017).

At present, the most prominent approach to control DM involves the use of oral synthetic hypogycemic drugs such as sulphonylureas, biguanide, α-glucosidase, and dipeptidyl peptidase-4 (DPP-4) inhibitors. However, these drugs have characteristic profiles of short- and/or long-term side effects, which include hypoglycemia, weight gain, gastrointestinal discomfort and nausea, liver and heart failure (Hung et al., 2012). Additionally, the drugs are costly in the developing countries especially in Asia and African regions. These limitations have prompted the search for potent plant-derived bioactive ingredients as possible alternative therapies for DM. The target is to identify newer compounds that could attenuate hyperglycemia, ameliorate the diabetes associated-complications with fewer adverse effects. These can be standardized and used as the drugs for the treatment of DM.

Spices add flavor, taste, and color in food preparation and most importantly, consumption of spices provide infinite health benefits to humans. Considerable evidence has shown that spices play a vital role in ameliorating DM complications and were documented in several reviews (Khan and Safdar, 2003; Kelble, 2005; Srinivasan, 2005; Mohamed, 2014; Kazeem and Davies, 2016; Bi et al., 2017). However, most of the available reviews focused on the extracts derived from the spices. Although some of the reviews highlighted the hypoglycemic roles of the bioactive ingredients derived from the spices (Upaganlawar et al., 2013; Zhang et al., 2013; Semwal et al., 2015), the critical assessment of their hypoglycemic potency based on the concentration/dose has not yet been well documented. The exaggerations of the data obtained from *in vitro* and *in vivo* studies are of concerns. In other words, whether these active ingredients are potential hypoglycemic agents or adjuvants, not clear at all. On the other hand, the lack of bioavailability is the major factor affecting the overall bioactivity of the spice-derived bioactive ingredients (SDBI) (Huang et al., 2010; Yao et al., 2015). Therefore, we intended to comprehensively review all the published studies on the hypoglycemic action of SDBI with critical assessment whether the ingredients are potential hypoglycemic agents or adjuvants. In addition, future prospects, safety and the progress made on the methods used to improve the bioavailability of the promising SDBI were included in this review as well.

## Methodology

**Figure 1:**
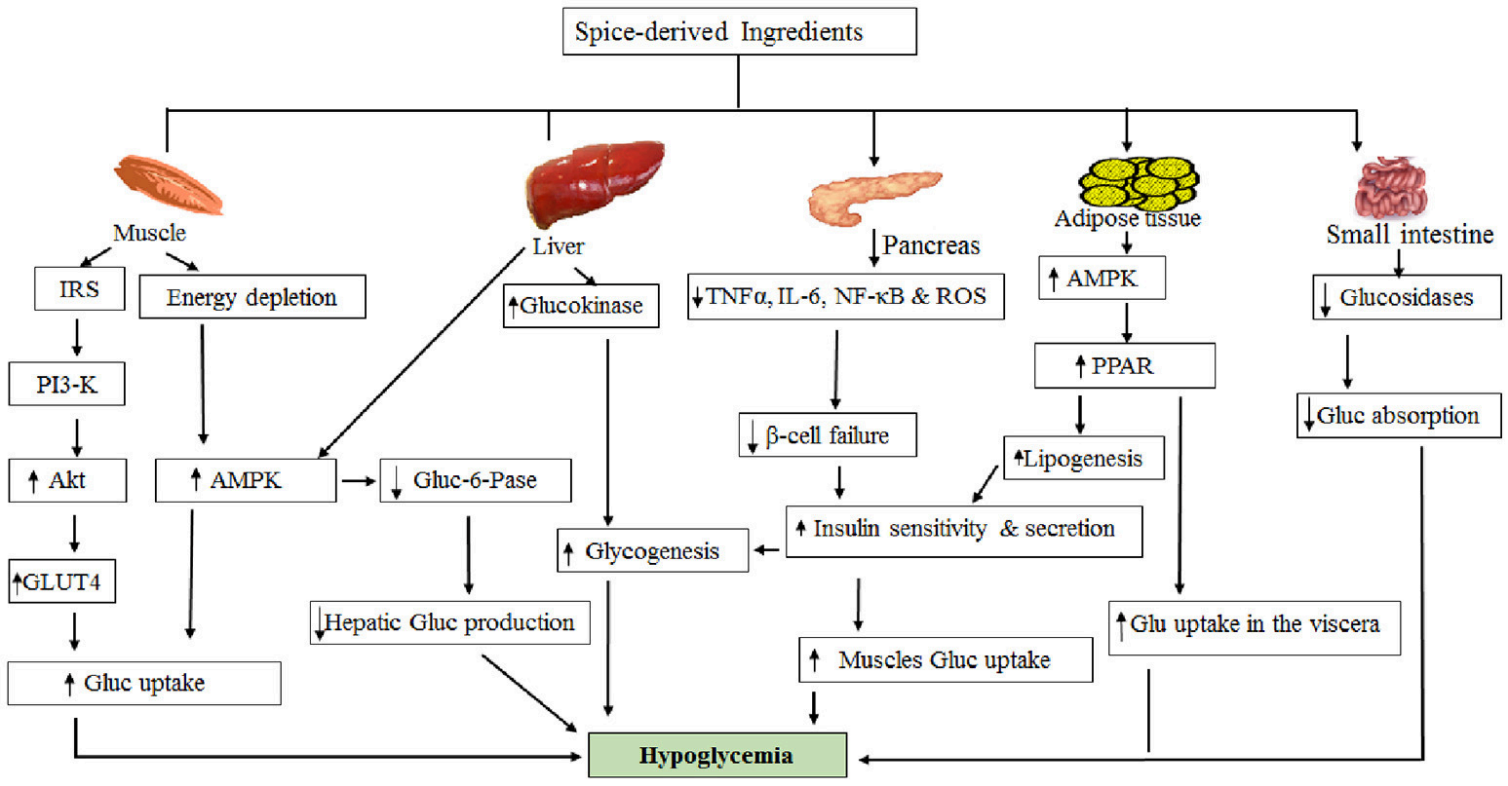
Possible mechanism of hypoglycemic action by spice-derived ingredients.

In the present study, we considered the SDBI as potential hypoglycemic agents based on multiple citations that showed >50% blood glucose reduction potential at non-toxic dosages. The potent hypoglycemic action using *in vitro* models (lower IC_50_ values) and less toxicity associated with the target compounds were also considered in this study. The hypoglycemic roles of the SDBI were categorized and presented based on *in vitro* (Table 1), *in vivo* (Supplementary Table 1) or clinical (Table 2) studies. Additionally, proposed hypoglycemic mechanisms depicted by the promising SDBI are presented in Figure 1.

**Table 1:** *In vitro* studies of spice-derived ingredients.

**Compounds**	**Dosages**	**Efficacy**	**References**
Diallyl trisulfide	1–10 μM	Suppresses hyperglycemia-induced cardiomyocyte apoptosis in H9c2 cells	Kuo et al., 2013
Capsaicin	140 μg/ml	Inhibits intestinal glucose transport in isolated rats muscles	Monsereenusorn and Glinsukon, 1978
	20–250 μM	Inhibits hyperlipidemia in 3T3-L1 adipocytes	Hwang et al., 2005; Berkoz et al., 2015
	0–250 μM	Inhibits hyperlipidemia in 3T3-L1 pre-adipocytes and adipocytes	Hsu and Cheng, 2007
	0.1–10 μM	Stimulates lipolysis in differentiated 3T3-L1 adipocytes	Lee M. S. et al., 2011
	5–1,000 μg/ml	Inhibits α-amylase and α-glucosidase actions	Tundis et al., 2013
	50, 100 μM	Increases glucose uptake in C2C12 muscle cells	Kim et al., 2013
Isodihydrocapsiate (capsaicinoid-like substance)	30–100 μM	Stimulates plasma glucose uptake in L6 myotubes	Hwang et al., 2008
Cinnamaldehyde	0.5–500 μg/100 mL	Inhibits of aldose reductase activity	Lee, 2002, 2005
	–	Inhibits of α-glucosidase activity	Lee, 2005
	0.1–100 μM	Impairs high glucose-induced hypertrophy in NRK-49F- renal interstitial fibroblasts	Chao et al., 2010
	10–40 μM	Down-regulates the expression of PPARγ in 3T3-L1 pre-adipocytes	Huang et al., 2011
	2.5–10 μM	Down-regulates iNOS and COX2 gene expression	Yuan et al., 2011
	10–50 μM	Up regulates the expression of GLUT4 gene in C2C12 mouse skeletal muscle	Nikzamir et al., 2014
	50–200 μM	Promotes glucose-stimulated insulin release in isolated rat islets	Hafizur et al., 2015
Curcumin			
	5 μM	Attenuates lipopolysaccharide (LPS)-induced production of TNFα in human monocytic macrophage cells	Chen, 1995
	20 μM	Mimics insulin action in hepatic stellate cells	Zheng and Chen, 2004
	0–10 μM	Prevents glycosylation in human erythrocytes cells	Jain et al., 2006
	1–25 μM	Supresses insulin-induced HSC activation	Masamune et al., 2006
	2–10 μM	Stimulates β-cell function in isolated rat pancreas	Best et al., 2007
	10–80 μM	Antioxidative in isolated STZ-induced C57/BL6J diabetic mice	Meghana et al., 2007
	20–80 μM	Protects pancreatic islets against cytokine-induced cell death	Kanitkar et al., 2008
	2–200 μM	Inhibits hepatic gluconeogenesis and glycogenolysis in isolated mice hepatocytes and hepatoma cells	Fujiwara et al., 2008; Kim et al., 2009
	10–60 μM	Decreases TNF-α, IL-6, IL-8, and MCP-1 secretion in high glucose-treated cultured monocytes	Jain et al., 2009
	5–20 μM	Improves insulin sensitivity in 3T3-L1 adipocytes	Wang et al., 2009
	5–30 μM	Supresses insulin-induced HSC activation in type I collagen gene	Lin et al., 2009
		Inhibits glycogen synthase kinase-3β activity (IC_50_: 66.3 nM)	Bustanji et al., 2009
	0.01–1 μM	Increases glucose uptake in isolated rat skeletal muscle	Cheng et al., 2009
	3–60 μM	Stimulates glucose uptake in C2C12 and L6 myotube cells	Kang and Kim, 2010; Kim et al., 2010
	10–60 μM	Stimulates glucose uptake in L6 myotube cells	Kim et al., 2010
	0–30 μM	Antihyperglycemic	Lin and Chen, 2011
	2.5–30 μM	Suppresses the lipolysis in 3T3-L1 adipocytes	Xie et al., 2012
	10–100 μg/ml	Inhibits α-amylase activity	Satapathy and Panda, 2013
	0–100 μM	Inhibits glucose transport in 3T3-L1 adipocytes	Green et al., 2014
	1–100 pM	Enhances pancreatic β-cell function in human pancreatic islet β-cells	Rouse et al., 2014
Turmerone	–	Inhibits α-amylase; α-glucosidase actions	Lekshmi et al., 2012a
Turmerin	–	Inhibits α-amylase; α-glucosidase actions	Lekshmi et al., 2012b
Diosgenin	0.33, 3.3 mg/ml	Inhibits glucose uptake in isolated intestinal rabbits	Al-Habori et al., 2001
	1–10 μM	Enhances glucose uptake in 3T3-L1 cells.	Uemura et al., 2010
	0.1–10 μM	Attenuates insulin resistance in HUVE cells	Liu et al., 2012
	0.5–10 μM	Suppresses dyslipidemia in 3T3-L1 preadipocytes	Sangeetha et al., 2013
	100 μg/ml	Inhibits α-amylase and α-glucosidase activity	Ghosh et al., 2014
Eugenol	0–100 μM	Increases the expressions of GLUT4 and PI3K genes in L6 myotubes	Prabhakar and Doble, 2011
	–	Inhibits α-amylase; lipase; angiotensin converting enzyme actions	Mnafgui et al., 2013
	5–20 μM	Antihyperglycemic in SHSY5Y cells	Prasad et al., 2015
	0–30 mM	Inhibits advanced glycation end products	Singh et al., 2016
Galactomannan	200 mg/ml	Inhibits α-amylase actions	Kashef et al., 2008
	0.1, 0.5% w/w	Inhibits intestinal glucose uptake in isolated intestine of lean and obese rats	Srichamroen et al., 2009
	-	Promotes glucose uptake in hemidiaphragm of treated alloxanized rats	Anwar et al., 2009
[6]-Gingerol	25 μM	Inhibits TNF-α mediated downregulation of adiponectin expression in 3T3-L1 adipocytes	Isa et al., 2008
	2.5–20 μM	Attenuate β-amyloid-induced oxidative cell death in SH-SY5Y neuroblastoma cells	Lee C. et al., 2011
	25, 50, 100, 150 μM	Enhances glucose uptake in L6 myotubes	Li J. et al., 2012; Li et al., 2013; Son et al., 2015
	10 μM	Prevents diastolic dysfunction in isolated murine ventricular myocardia	Namekata et al., 2013
	0–30 μM	Stimulates glucose uptake in L6 and C2C12 cells	Lee et al., 2015
	6.25–50 μM	Inhibits lipid accumulation in 3T3-L1 adipocytes	Tzeng and Liu, 2013; Tzeng et al., 2014; Son et al., 2015; Choi et al., 2016
	30–240 μg/ml	Inhibits α-amylase; α-glucosidase activity	Mohammed et al., 2017
[6]-Shogaol	25 μM	Inhibits TNF-α mediated downregulation of adiponectin expression in 3T3-L1 adipocytes	Isa et al., 2008
	100 μM	Promotes glucose utilization in 3T3-L1 adipocytes and C2C12 myotubes	Wei et al., 2017
	30–240 μg/ml	Inhibits α-amylase and α-glucosidase activity	Mohammed et al., 2017
[6]-Paradol	100 μM	Promotes glucose utilization in 3T3-L1 adipocytes and C2C12 myotubes	Wei et al., 2017
	30–240 μg/ml	Inhibits α-amylase and α-glucosidase activity	Mohammed et al., 2017
	0–160 μM	Inhibits adipogenesis in 3T3-L1 adipocytes	
4-Hydroxyisoleucine	10 μM to 1 mM	Stimulates insulin released in isolated rat pancreas and L6 myotubes	Sauvaire et al., 1998; Broca et al., 2000; Wang et al., 2002; Rawat et al., 2014
	5–25 μM	Stimulates glucose uptake in L6-GLUT4 *myc* myotubes	Jaiswal et al., 2012
	5–25 μM	Ameliorates insulin resistance and shows anti-inflammatory activity in L6 myotubes	Maurya et al., 2014
	5–25 μM	Stimulates glucose uptake and insulin release in L6 skeletal muscle cells	Korthikunta et al., 2015
	100 ng/mL	Stimulates proximal insulin signaling, Increases expression of glycogenic enzymes and GLUT2 in HepG2 cells	Naicker et al., 2016
Piperine	10–5,000 μg/ml	Inhibits α-lipase, α-glucosidase and aldose reductase activities	Kumar P. T. et al., 2013
Thymoquinone	3 mg/kg	Anti-inflammatory in isolated STZ-induced peritoneal macrophages	El-Mahmoudy et al., 2005a
	2.5 μM	Promotes glucose stimulated insulin secretion in rat pancreatic β-cells	Chandra et al., 2009
	10–50 μM	Shows antiglycation activity	Losso et al., 2011; Anwar et al., 2014; Khan et al., 2014
	0–5 μM	Improves insulin secretion from pancreatic β-cells in INS-1 cells	Gray et al., 2016
Trigonelline	0.33, 3.3 mg/ml	Inhibits glucose uptake in isolated intestinal rabbits	Al-Habori et al., 2001
	25–100 μM	Hypolipidemic in 3T3-L1 cells	Ilavenil et al., 2014

**Table 2:** Clinical trials of spice-derived ingredients.

**Compounds**	**Dosages/periods**	**Efficacy**	**References**
Capsaicin	0.075% (topical)/4 days for 8 weeks	Ameliorates diabetic neuropathy in diabetic patients	Scheffler et al., 1991; Biesbroeck et al., 1994; Forst et al., 2002
	5 mg/day for 4 weeks	Antihyperlipidemic in women with gestational diabetes mellitus	Yuan et al., 2016
Curcumin	150 mg/twice daily for 8 weeks	Antihyperglycemic, Ameliorates insulin resistance	Usharani et al., 2008
	250 mg/day for 9 months	Antihyperglycemic, Ameliorates insulin resistance	Chuengsamarn et al., 2012, 2014
	475 mg/day for 10 days	Antihyperglycemic, Antihyperlipidemic in type 2 diabetic patients	Neerati et al., 2014
Trigonelline	500 mg/day after 2 h	Improves glucose tolerance in overweight men	Van Dijk et al., 2009

### *S*-Allyl cysteine and its derivatives

#### *In vitro* studies

Diallyl trisulfide (DATS) an organosulfur from garlic (*Allium sativum* L.) at various concentrations (1–5 μM) suppressed high glucose-induced cardiomyocyte apoptosis via inhibition of NADPH oxidase, reactive oxygen species (ROS) production and downregulated JNK/NF-κB signaling in H9c2 cells (Kuo et al., 2013). This shows the potential of DATS in the management of diabetes-associated inflammation.

#### *In vivo* studies

Saravanan and colleagues have shown that oral administration of *S*-Allyl cysteine (SAC) treatment at 150 mg/kg bw for 45 days reduced fasting blood glucose (FBG) by 65%, ameliorated oxidative damages, glycosuria and improved the activities of glucose metabolizing enzymes in STZ-diabetic rats (Saravanan et al., 2009, 2010, 2013; Saravanan and Ponmurugan, 2010, 2011, 2012a,b). Oral supplementation of SAC (0.5–1.0 g/l) for 4 or 10 weeks showed 34% more FBG reduction compared to *n*-acetyl cysteine, *S*-ethyl cysteine, *S*-methyl cysteine, and *S*-propyl cysteine (<30% FBG reduction) in STZ-induced Balb/cA mice (Hsu et al., 2004; Mong and Yin, 2012). Additionally, SAC was shown to have potent protection against renal inflammation via suppressing NF-κB activity and NF-κB p65 mRNA expression in STZ-induced diabetic rats (Mong and Yin, 2012).

Oral administration of alliin [*S*-allyl cysteine sulfoxide (SACS)] and *S*-methyl cysteine sulfoxide (SMCS) at 200 mg/kg bw for 30 days decreased FBG by 44.5 and 38%, respectively in alloxan-induced diabetic rats (Sheela et al., 1995). Furthermore, alliin, a sulfoxided from garlic, decreased serum glycosylated hemoglobin, the activities of phosphatases, lactate dehydrogenase and glucose-6-phosphatase enzymes and increased serum insulin level, liver and intestinal HMG-CoA reductase and hexokinase activities in alloxan-induced diabetic rats (Sheela and Augusti, 1992; Augusti and Sheela, 1996). Conversely, consumption of diallyl diasulfide (DADS) and DATS (40–80 mg/kg bw) for 16 or 3 weeks showed no effects on FBG in STZ-induced diabetic rats (Liu et al., 2005, 2006). Interestingly, treatment of DATS (40 mg/kg bw) for 16 days reduced the expression of phosphorylated JNK and NF-κB, and active caspase 3 in cardiac myocytes of STZ-induced diabetic rats (Kuo et al., 2013). This supports the *in vitro* data published by Kuo et al. (2013) and further showed DATS ability to ameliorate diabetes-induced elevation of inflammatory mediators such as tumor necrosis factor-alpha (TNFα) in the muscles. Additionally, oral administration of *S*-allyl-mercapto-captopril (alliin and Captopril conjugate) at 53.5 mg/kg bw for 55 days reduced FBG (42%) and blood pressure in Cohen-Rosenthal diabetic hypertensive rats (Younis et al., 2010). Allicin (derived from hydrolysis of alliin) at 250 mg/kg bw decreased blood glucose levels and improved glucose tolerance after 4 h post-administration period in alloxan-induced diabetic rabbits (Mathew and Augusti, 1973).

#### Toxicity

Based on the present literature search, studies on the detail toxicities associated with organosulfur compounds under study are scanty. However, Rao and Natarajan (1949) reported the subcutaneous and intraperitoneal LD_50_ of allicin are 5 and 20 mg/kg bw, respectively.

#### Recommendation

According to the above-mentioned studies, the spice-derived sulfur containing ingredients showed their hypoglycemic effects not only by decreasing FBG, oxidative stress, inflammatory biomarkers but also by increasing insulin secretion and improving glucose tolerance and glucose metabolism-related enzyme activities. However, based on the levels of hypoglycemic potential of sulfur containing compounds and their derivatives, these compounds (SAC, SMCS, SACS, DADS, DATS, and allicin) cannot be considered as hypoglycemic agents but only as adjuvants.

### Capsaicin

#### *In vitro* studies

The hypoglycemic action of capsaicin from *Capsicum* species seems to be controversial and contradictory. Monsereenusorn and Glinsukon (1978) have reported that capsaicin (140 μg/ml) inhibited intestinal glucose transport (22.6%) mediated by GLUT2, attributed to the Na^+^-K^+^-ATPase pump (Monsereenusorn and Glinsukon, 1979). In another study, capsaicin (5–1,000 μg/ml) exhibited α-amylase (IC_50_: 83 μg/ml) and α-glucosidase (IC_50_: >500 μg/ml) inhibitory activities (Tundis et al., 2013), implying a possible role in ameliorating post-prandial hyperglycemia. In addition, capsaicin and its derivative (isodihydrocapsiate) at various concentrations (50–100 μM) stimulated glucose uptake, via AMP-activated protein kinase (AMPK) up regulation in C2C12 muscle or L6 myotube cells, respectively (Hwang et al., 2008; Kim et al., 2013). Moreover, capsaicin (0–250 μM) inhibited lipid accumulation in 3T3 L1 pre-adipocytes and adipocytes, implying the role of capsaicin in attenuating insulin resistance (Hsu et al., 2004; Hwang et al., 2005; Lee M. S. et al., 2011).

#### *In vivo* studies

Intraperitoneal treatment of capsaicin (20–50 mg/kg bw) for 9 weeks attenuated hyperglycemia (44% reduction), improved glucose homeostasis and insulin release in Zucker diabetic fatty (ZDF) rats and partial pancreatectomized diabetic rats (Gram et al., 2007; Kwon et al., 2013). Accordingly, dietary inclusion of capsaicin (0.015%) for 3 weeks decreased hyperglycemia (17%) and ameliorated dyslipidemia, inflammation and insulin resistance in KK-A^y^ obese/diabetic mice, which linked to its dual action on PPAR-α and TRPV-1 expression/activation (Kang et al., 2011a,b). Similarly, capsaicin (0.0024–0.0042%) inclusion in diet showed maximum FBG reduction of 49% in the same animal model (Okumura et al., 2012).

Notably, administration of capsaicin (10 μg/kg bw) for 20 weeks prevented the onset of type 1 diabetes in a non-obese diabetic mouse model, attributed to the attenuation of antigen-specific T-cells in pancreatic lymph nodes (Nevius et al., 2012). Conversely, animals treated with other dosages (0.1, 1.0, 25.0, and 50.0 μg/kg bw) were hyperglycemic throughout the study period, which is a subject for further studies. More recently, dietary inclusion of capsaicin (0.014–0.1%) for 12 weeks decreased serum and tissue advanced glycation end products (AGEs) and activated the receptor for AGEs (RAGE) in STZ-induced diabetic rats (Hsia et al., 2016). However, the reduction of FBG in the capsaicin-treated groups was not significant compared to the diabetic untreated group (Hsia et al., 2016). This further supports the previous studies that capsaicin administration (0.015%) has no effect on the blood glucose level in the same animal model (Babu and Srinivasan, 1997a; Suresh Babu and Srinivasan, 1998). Furthermore, the dietary inclusion of capsiate (0.025%/7 weeks), a non-pungent capsaicin analog, improved glucose tolerance ability (28%) via improving insulin sensitivity in pancreatectomized diabetic rats (Kwon et al., 2013).

#### Clinical trials

In a randomized, double-blind, placebo-controlled trial, oral administration of capsaicin (5 mg/day) for 4 weeks attenuated insulin resistance and dyslipidemia with no significant effect on FBG in women with gestational diabetes (Yuan et al., 2016). In addition, topical application of capsaicin (0.075%) for 8 weeks ameliorated painful diabetic neuropathy in diabetic patients (Scheffler et al., 1991; Tandan et al., 1992; Biesbroeck et al., 1994; Forst et al., 2002).

#### Toxicity

The oral LD_50_ values of capsaicin were within the ranges 90–162 mg/kg bw for mice and rats (Saito and Yamamoto, 1996). However, the intraperitoneal, intravenous, and subcutaneous LD_50_ values for mice were 7.65, 0.56, and 9 mg/kg bw in mice, indicating possible toxicity (Glinsukon et al., 1980). To further support this, some adverse consequences of capsaicin consumption reported include nausea, vomiting, abdominal pain, burning diarrhea, intense tearing and conjunctivitis (Goldfrank, 2002; Millqvist et al., 2005). Additionally, Marques et al. (2002) have reported that people consuming capsaicin (90–250 mg/day) are more susceptible gastrointestinal cancer compared to the subjects consumed lesser doses of capsaicin (0–29.9 mg/day).

#### Recommendation

From the above-mentioned studies, although capsaicin showed mild to moderate hypoglycemic activity by inhibiting glucose digesting enzymes activities, improving glucose uptake, decreasing insulin resistance, dyslipidemia, advanced glycation endproducts; the reduction of FBG and hyperglycemia was not promising. Therefore, it may not be a good candidate for DM therapy. Our argument is that none of the studies reported >50% reduction of blood glucose levels despite several weeks of administration. Additionally, capsaicin consumption showed weak antihyperglycemic effect in women with gestational diabetes (Yuan et al., 2016). The toxicities associated with capsaicin consumptions are another great concern. Despite intraperitoneal administration conferred higher action compared to the oral administration, it was more susceptible to adverse consequences and hence should be discouraged. However, capsaicin topical application is encouraged to reduce some complications associated with diabetic neuropathy as this was validated in some clinical trials (Scheffler et al., 1991; Tandan et al., 1992; Biesbroeck et al., 1994; Forst et al., 2002). This justified the use of capsaicin as adjuvant in the management of DM, particularly diabetic neuropathy.

### Cinnamaldehyde

#### *In vitro* studies

Cinnamaldehyde is an aromatic aldehyde and main bioactive component of cinnamon (*Cinnamomum zeylanicum* var. *cassia* Meisn.). Several studies have reported the potential of cinnamaldehyde in the prevention of diabetes related-complications. Lee (2005) reported that cinnamaldehyde (0.005–5 μg/ml) is a potent aldose reductase (IC_50_: 0.8 μg/ml) and weak α-glucosidase (IC_50_: 500 μg/ml) inhibitor signifying its potential in attenuating osmotic imbalance in non-insulin dependent tissues and hence, ameliorated diabetic retinopathy.

Cinnamaldehyde (10–50 μM) attenuated lipid accumulations in 3T3 preadipocytes via PPARδ, PPARγ, AMPK, and retinoid X receptor (RXR) expression. and thus, helps to prevent insulin resistance (Huang et al., 2011; Li et al., 2015). Similarly, cinnamaldehyde (2.5–10 μM) prevented STZ-induced pancreatic β-cell damage in RINm5F rat insulinoma cells (Yuan et al., 2011). This effect was linked to the downregulation of iNOS and COX-2 genes expression through blocking the NF-κB and MAPKs activities that ultimately prevented pancreatic ROS elevation and damage. Chao et al. (2010) reported that cinnamaldehyde (0.1–100 μM) reduced high glucose-induced hypertrophy in NRK-49F- renal interstitial fibroblasts through inactivation of the p38 MAPK pathway, linked to diabetic nephropathy. Nikzamir et al. (2014) have demonstrated that cinnamaldehyde (10–50 μM) stimulated glucose transporter 4 (GLUT4) gene expression in C2C12 mouse skeletal muscle. Hafizur et al. (2015) have shown the potential of cinnamaldehyde to induce glucose-stimulated insulin release in isolated islets, which could facilitate glucose transport into the cells and thus reduced hyperglycemia.

#### *In vivo* studies

Oral administration of cinnamaldehyde (5–20 mg/kg bw) for 45 days reduced FBG (63.3%), lipid accumulation and showed insulinotropic action in STZ-induced diabetic rats (Subash Babu et al., 2007). Interestingly, the same authors have recently reported a more potent FBG reduction (71%) while used the same doses, study period and animal models (Subash Babu et al., 2014). The potent antihyperglycemic action of cinnamaldehyde was linked to the upregulation of GLUT4 protein expression that may facilitate the transport of glucose across the cells (Zhang et al., 2008; Anand et al., 2010; Jawale et al., 2016). Importantly, Zhang et al. (2008) showed a 62% reduction of FBG and improved insulin sensitivity in pancreatic β-cell upon cinnamaldehyde (40 mg/kg bw) consumption in a high-fat diet-fed STZ-induced diabetic rat model. This was supported even at a lower dosage of cinnamaldehyde (143.8 μmol/kg bw) for 4 weeks in high-fat-diet-induced insulin resistant rats (Farrokhfall et al., 2014), and thus, corroborates with the *in vitro* studies (Huang et al., 2011; Li et al., 2015).

Treatment of cinnamaldehyde (20 mg/kg bw) for 6 weeks curtailed FBG (40%), insulin resistance and diabetes-induced hypertension in STZ-induced diabetic rats, attributed to the restoration of vascular contractility in the treated rats (El-Bassossy et al., 2011). In the same animal model, oral gavage of cinnamaldehyde (20 mg/kg bw) reduced FBG by 21.1 and 69.8% after 4 h and 4 weeks post-treatment period, respectively and ameliorated diabetes-induced alterations (Kumar et al., 2012). Subsequently, administration of cinnamaldehyde (20 mg/kg bw) for 4 weeks was shown to attenuate hyperglycemia, TNF-α mRNA expression and upregulated GLUT-4 mRNA expression in C57BLKS/J db/db mice (Li J. et al., 2012; Guo et al., 2017). In fatty-sucrose diet/streptozotocin (FSD/STZ)-rat model of gestational diabetes, supplementation of cinnamaldehyde (25 mg/kg bw) for 8 weeks reduced FBG (80%) via modulation of PPARγ, proinflammatory cytokines and oxidative stress (Hosni et al., 2017).

Ghrelin a hunger hormone, participate in the regulation of glucose and insulin metabolism. The plasma ghrelin levels are shown to correlate inversely with insulin levels and are associated with insulin resistance and could be a potential target to reduce the progression of type 2 diabetes (Pulkkinen et al., 2010; Tong et al., 2010). Conforming to this, dietary inclusion of cinnamaldehyde (0.2%) for 36 days retarded the endogenous ghrelin release and reduced FBG (10%) in C57BL6 diabetic mice (Camacho et al., 2015).

#### Toxicity

The low toxicity associated with cinnamaldehyde consumption in rodents via oral route has been well documented (Jenner et al., 1964; Sporn et al., 1965; Zaitsev and Rakhmanina, 1974; Subash Babu et al., 2007). Seemingly, Hooth et al. (2004) reported that the safety of cinnamaldehyde was approved by the Food and Drug Administration (FDA) and has been given Generally Recognized as Safe (GRAS) status in the United States. However, Weibel and Hansen (1989) have reported that cinnamaldehyde elicits some carcinogenic risk by acting as an alkylating agent that could react with cellular macromolecules.

#### Recommendation

According to the results of the above-mentioned studies, cinnamaldehyde is a potential hypoglycemic agent and adjuvant. Several studies have shown that cinnamaldehyde reduced FBG by >50% at 20 or 40 mg/kg bw in various animal models (Subash Babu et al., 2007, 2014; Zhang et al., 2008; Kumar et al., 2012). Regarding the *in vitro* studies, this ingredient showed potent hypoglycemic potential at <10 μg/ml or μM and depicted IC_50_ values of <10 μg/ml as well (Yuan et al., 2011; Kumar et al., 2012). These are of interest in the drug discovery as small amount of the compound stimulated beneficial action in various models. Additionally, the less toxicity associated with cinnamaldehyde intake is of significance in drug design and development. However, the lack of clinical trials with cinnamaldehyde is a major drawback in determining its exact hypoglycemic potential in human subjects. Hence, further studies, particularly clinical studies, are warranted to confirm the hypoglycemic effects of cinnamaldehyde in humans.

### Curcumin

#### *In vitro* studies

Curcumin is the major active principle of turmeric (*Curcuma longa* L.) and has been reported to possess tremendous potential including hypoglycemic action. Several studies have shown that curcumin (20 μM) stimulated insulinotropic action via PPARγ activation and attenuated oxidative stress in hepatic stellate cells (Zheng and Chen, 2004; Masamune et al., 2006; Lin et al., 2009; Lin and Chen, 2011). Jain et al. (2006, 2009) have reported that curcumin (0–40 μM) prevents glycation, decreased TNF-α, IL-6, IL-8, and MCP-1 secretion in isolated human erythrocytes and high glucose-treated cultured monocytes. Furthermore, curcumin (20–80 μM) protected pancreatic islets against cytokine-induced cell death via scavenging ROS and decreased cytokine induced NF-kB translocation (Kanitkar et al., 2008). These studies have shown that amelioration of oxidative stress could be among the possible mechanism of curcumin hypoglycemic action. Furthermore, curcumin (2–40 μM) improved glucose absorption by activating the volume-regulated anion channel in isolated pancreatic β-cells and C2C12 mouse myoblast cells (Best et al., 2007; Kang and Kim, 2010). Curcumin (2–200 μM) was reported to activate AMPK and suppress gluconeogenic enzymes gene expression in hepatoma cells, which indicates the blood glucose lowering ability of curcumin (Kim et al., 2009, 2010). To support this, curcumin (25 μM) inhibited hepatic gluconeogenesis and glycogenolysis in isolated mice hepatocytes (Fujiwara et al., 2008).

Curcumin (5–20 μM) was shown to improve insulin sensitivity in 3T3-L1 adipocytes (Wang et al., 2009), which is linked to the suppression of lipolysis and inhibition of glucose transport (Xie et al., 2012; Green et al., 2014). Increased glycogen synthase kinase-3β activity has been implicated in type 2 diabetes insulin resistance, mediated via phosphatidylinositol kinase-3 activation and the inhibition of protein kinase B (Pandey and DeGrado, 2016). Bustanji et al. (2009) have reported that curcumin inhibited glycogen synthase kinase-3β activity (IC_50_: 66.3 nM). In a more recent study, curcumin (10–100 μg/ml) inhibited α-amylase action (Satapathy and Panda, 2013). Curcumin (0.01–60 μM) was shown to stimulate glucose uptake in isolated rat skeletal muscle and in L6 myotube cells (Cheng et al., 2009). Additionally, curcumin (1 pM−80 μM) was shown to enhances pancreatic β-cell function in isolated human pancreatic islets (Meghana et al., 2007; Rouse et al., 2014).

#### Turmerone and turmerin

Lekshmi et al. (2012a) have reported that turmerone from turmeric exhibited potent α-amylase (IC_50_: 24.5 μg/ml) and α-glucosidase (IC_50_: 0.28 μg/ml) inhibitory actions. In another study, turmerin, a water-soluble peptide in turmeric rhizomes, was reported to show α-amylase (IC_50_: 192 μg/ml) and α-glucosidase (IC_50_: 31 μg/ml) inhibitory actions as well (Lekshmi et al., 2012a). These ingredients have demonstrated the potential in reducing post-prandial hyperglycemia in diabetes.

#### *In vivo* studies

Several studies have reported the hypoglycemic effect of curcumin using various animal models. Babu and Srinivasan (1997b); Suresh Babu and Srinivasan (1998) have shown that dietary supplementation of curcumin (0.5%) for 8 weeks attenuated hyperlipidemia and renal dysfunction in STZ-induced diabetic rats. Conversely, the authors reported no reduction on the FBG levels in the treated diabetic rats which is consistent with some previous studies (Suryanarayana et al., 2007; Palma et al., 2014). However, the above-mentioned studies have reported potent antioxidant action in the same model which is in line with some previous studies (Sajithlal et al., 1998; Rungseesantivanon et al., 2010; Gupta et al., 2011). These effects of curcumin are in line with the results of *in vitro* studiesas presented above (Zheng and Chen, 2004; Jain et al., 2006, 2009; Masamune et al., 2006; Kanitkar et al., 2008; Lin et al., 2009; Lin and Chen, 2011).

Oral administration of curcumin (80–100 mg/kg bw) for 3 or 7 weeks reduced FBG (31.4%) and serum glycated hemoglobin (30.6%) in alloxan-induced diabetic rats (Arun and Nalini, 2002). Dietary intervention of curcumin (0.001–0.005% w/v) for 8 weeks delayed the progression of cataract via the downregulation of vascular endothelial growth factor (VEGF) expression in STZ-induced diabetic rats (Suryanarayana et al., 2005; Kowluru and Kanwar, 2007; Mrudula et al., 2007). Supplementation of curcumin (0.5%) for 2 weeks decreased bone resorptive activity via attenuating osteoclastogenesis in STZ-induced diabetic rats (Hie et al., 2009).

Dietary supplementation of curcumin (0.02%) for 6 weeks decreased FBG (22%) in C57BL/KsJ-db/db diabetic mice (Seo et al., 2008). In KKAy diabetic mice, dietary inclusion of curcumin at 0.24% for 5 weeks increased hepatic glycolysis and overall lipids metabolism, which might help in reducing the hyperglycemia (Honda et al., 2006). Kanitkar et al. (2008) have reported that oral treatment of curcumin (7.5 mg/kg bw) for 5 days reduced FBG (69%) and ameliorated pancreatic β-cell damage in STZ-induced diabetic mice. In a series of studies, curcumin (10 or 80 mg/kg bw) treatment for 45 days showed maximum FBG reduction of 57.1%, antihyperlipidemic, insulinotropic and antioxidant activities in type 1 and type 2 diabetic rat models (Murugan and Pari, 2006a,b, 2007; Pari and Murugan, 2007b; Murugan et al., 2008; Hussein and Abu-Zinadah, 2010; Abdel Aziz et al., 2013). Oral administration of photo-irradiated curcumin (10–80 mg/kg bw) for the same period reduced FBG (53.9%) and ameliorated lipid peroxidation in STZ-induced diabetic rats (Mahesh et al., 2004, 2005). This imply that photo-irradiation has no effect on the hypoglycemic action of curcumin, since the reductions of FBG by photo-irradiated (53.9%) or non-photo-irradiated (57.1%) curcumin were not significantly different.

Oral administration of curcumin (15 or 30 mg/kg bw) for 6 weeks reduced FBG (24.4%) and attenuated renal dysfunction at the maximum dosage administered in STZ-induced diabetic rats (Sharma et al., 2006). Similarly, consumption of curcumin (60 mg/kg bw) for 2 weeks to the same animal model improved brain stem function attributed to the regulations of cholinergic, insulin receptor and GLUT-3 in the brain stem (Peeyush et al., 2009; Kumar P. T. et al., 2013). Curcumin treatment for 10 weeks ameliorated hyperglycemia (44.3%), cognitive deficit, cholinergic dysfunction, oxidative stress and inflammation in the same animal model and dosage (Kuhad and Chopra, 2007). Furthermore, Awasthi et al. (2010) have reported that oral administration curcumin (10–50 mg/kg bw) for 3 weeks provented intracerebral STZ-induced impairment in memory and cerebral blood flow. Chiu et al. (2009) showed that curcumin treatment (150 mg/kg bw) for 4 weeks reduced FBG and downregulated the expression of p300 and nuclear factor-κB in STZ-induced diabetic rats. Oral administration of curcumin (200 mg/kg bw) for 2 weeks demonstrated anticholinesterase and antioxidant actions and attenuated diabetes-induced dementia in rats (Agrawal et al., 2010; Chanpoo et al., 2010; Mahfouz, 2011). This is in line with the previous data that curcumin protects pancreatic islets from cytokine-induced cell death via scavenging ROS and decreasing cytokine-induced NF-kB translocation (Kanitkar et al., 2008).

Curcumin treatment (60 mg/kg bw) downregulated β2-adrenoceptor gene expression and upregulated the insulin receptor gene expression in the muscles of STZ-induced diabetic rats, indicating decreased glycogenolysis, gluconeogenesis and increased glycogenesis in the muscles. (Xavier et al., 2012). Dietary inclusion of curcumin (0.5%) for 16 weeks improved the activities of lysosomal enzymes in liver, spleen, heart, lungs, testis and brain of STZ-induced rats (Chougala et al., 2012). El-Bahr (2013) have reported that oral administration of curcumin (15 mg/5 ml/kg bw) for 6 weeks to STZ-induced diabetic rats reduced FGB (43.7%) and improved the *in vivo* antioxidant status Consumption of curcumin (60 mg/kg bw) for 2 months to alloxan-induced diabetic rats decreased FBG and improved the pancreatic architecture to near normal (Acar et al., 2012; Abdel Aziz et al., 2013; Abdul-Hamid and Moustafa, 2013; Ghosh et al., 2015). Intraperitoneal administration of curcumin (10 mM) for 4 weeks reduced FBG (40%), exhibited pancreatic islet regenerative and antioxidative potential in STZ-induced diabetic rats (El-Azab et al., 2011). In some studies, oral administration of curcumin (100–200 mg/kg bw) for 2 or 8 weeks to STZ-induced diabetic rats ameliorated diabetic nephropathy and cardiomyopathy related symptoms (Soetikno et al., 2012, 2013; Zhao W. C. et al., 2014; Zheng et al., 2014). The proposed mechanism behind this effect was the inhibition of NADPH oxidase-mediating oxidative stress in the spinal cord and downregulation of the sphingosine kinase 1-sphingosine 1-phosphate (SphK1-S1P) signaling pathway (Soetikno et al., 2012; Huang et al., 2013).

Supplementation of curcumin (30–90 mg/kg bw) in yogurt for 31 days to STZ-induced diabetic rats showed antihyperglycemic and antihyperlipidemic actions (Gutierres et al., 2012). Rashid and Sil (2015) have shown that curcumin play a beneficial role against STZ-induced testicular abnormalities in diabetic rats. Consumption of curcumin (100 mg/kg bw) for 8 weeks reduced FBG (56.5%), intracellular Ca^2+^ level, active caspase cascade and the poly ADP-ribose polymerase (PARP) cleavage. Additionally, theNFκB-mediated inflammation was attenuated when the PI3K/Akt-dependent signaling was activated in the curcumin-treated animals (Rashid and Sil, 2015). This finding has suggested the protective role of curcumin against oxidative and ER stress in testes. Curcumin supplementation (50 or 100 mg/kg bw) for 3 weeks reduced hyperglycemia and the risk of vascular inflammation via attenuation of IL-6, MCP-1, TNF-α, HbA1, and lipid peroxidation in STZ-induced diabetic rats (Jain et al., 2009; Banafshe et al., 2014). In a nut shell, vast amount of data demonstrated curcumin to possess blood glucose and lipid-lowering abilities with subsequent improvement on insulin sensitivity in high fat-fed rats (Naito et al., 2002; Arafa, 2005; Kempaiah and Srinivasan, 2006; Jang et al., 2008; El-Moselhy et al., 2011; Na et al., 2011; Kaur and Meena, 2012; Hussein and El-Maksoud, 2013).

Administration of tetrahydrocurcumin (THC) a curcumin derivative (80 mg/kg bw) for 45 days reduced FBG (55%) and conferred potent antioxidant potential in STZ-induced diabetic rats (Karthikesan et al., 2010a,b). The effect was higher (67%) when co-administered with chlorogenic acid (5 mg/kg bw). This has indicated possible synergy with chlorogenic acid and warrant further study to understand the synergistic mode of interaction of THC and chlorogenic acid. Murugan and Pari have shown that administration of THC at the same dose and study period reduced FBG by 60% compared to 54.4% for curcumin (Pari and Murugan, 2005, 2007,b, 2008; Murugan and Pari, 2006a,b, 2007; Murugan et al., 2008). Additionally, a potent antihyperlipidemic, insulinotropic and antioxidant actions in diabetic rat models were also reported by the authors. This shows that the reduction of the FBG by the THC and curcumin is not significant and, Kanitkar et al. (2008) have reported a 69% reduction by the curcuim alone within short study period.

#### Clinical trials

Chuengsamarn and colleague reported that daily administration of curcumin at 250 mg for 6 and 9 months improved insulin action and lowered atherogenic risks in type 2 diabetic patients (Chuengsamarn et al., 2012, 2014). Previously, Usharani et al. (2008) reported that intake of curcumin capsules (150 mg) twice daily for 8 weeks to type 2 diabetic patients showed improved antioxidative status comparable to that of atorvastatin. Neerati et al. (2014) have recently reported that ingestion of curcumin (475 mg) for 10 day attenuated hyperglycemia and hyperlipidemia in type 2 diabetic patients. These studies compliment the *in vitro* and *in vivo* data despite lack of detail hypoglycemic potential in human subjects and signify the greater potential of curcumin in diabetes management.

#### Toxicity

Considerable amount of data is available, demonstrating curcumin safety and tolerability at the high doses (12 g/day) in several animal models (Lao et al., 2006a,b) and human subjects (Shankar et al., 1980; Chainani-Wu, 2003; Hsu and Cheng, 2007). However, some studies have shown that curcumin and its derivatives may cause hepatotoxicity, skin irritation and stomach ulcers when taken in high doses or for a prolonged period (Babu and Srinivasan, 1997b; Kandarkar et al., 1998; Balaji and Chempakam, 2010). Therefore, it is suggested that curcumin consumption at lower doses has no potential side effects. To further support this daily consumption of curcumin (500 mg) for 2 months was reported not to cause any adverse consequences in humans, except mild nausea and diarrhea (Hsu and Cheng, 2007; Chandran and Goel, 2012).

#### Recommendation

From the above-mentioned studies, it is evident that curcumin is the most investigated SDBI. Interestingly, numerous studies have reported FBG reduction of >50% with potent amelioration of diabetes-induced damages in various animal models without noticeable toxicity (Mahesh et al., 2004, 2005; Murugan and Pari, 2006a,b, 2007; Pari and Murugan, 2007b; Kanitkar et al., 2008; Murugan et al., 2008; Gutierres et al., 2012). To further support this, several *in vitro* studies have shown the potent curcumin hypoglycemic potential at concentrations even <10 μM (Best et al., 2007; Jain et al., 2006; Cheng et al., 2009; Wang et al., 2009; Kang and Kim, 2010). The less toxicity of curcumin intake in humans is encouraging and is of pharmacological interest as well.

### Diosgenin

#### *In vitro* studies

Diosgenin is a steroidal saponin and dietary ingredient from popularly consumed spice fenugreek (*Trigonella foenum-graecum* L.). Based on the current literature search, the information regarding the hypoglycemic potential of diosgenin *in vitro* is scanty. Liu et al. (2012) have reported that diosgenin (0.1–10 μM) attenuated insulin resistance associated endothelial dysfunction via inhibition of IKKβ and IRS-1 pathways in human umbilical vein endothelial cells (HUVECs). However, Fang et al. (2016) have recently linked the inhibition of insulin resistance to increase expression of the phosphorylated estrogen receptor-α (Erα), sarcoma (Src), Akt/protein kinase B and glycogen synthase kinase-3β (GSK-3β). The above data have demonstrated the diosgenin potential in amelioration of diabetes-associated insulin resistance.

In another study, diosgenin (0.5–10 μM) enhanced insulin-dependent glucose uptake and mitigate dyslipidemia via modulation of PPARs in 3T3-L1 preadipocytes (Uemura et al., 2010; Sangeetha et al., 2013). Diosgenin (100 μg/ml) showed uncompetitive mode of inhibition against α-amylase (70.9%) and α-glucosidase (81.7%) actions (Ghosh et al., 2014). Previously, diosgenin (0.33–3.3 mg/ml) was reported to inhibit glucose uptake (IC_50_: 8 mM) in isolated intestinal rabbits (Al-Habori et al., 2001). The above data suggest the beneficial role of diosgenin in controlling post-prandial hyperglycemia via delaying dietary glucose absorption and facilitating glucose uptake from the circulation.

#### *In vivo* studies

Dietary inclusion of diosgenin (10 g/kg bw) for 3 weeks reduced FBG (33.4%) and ameliorated dyslipidemia via modulation of Na^+^-K^+^-ATPase and increasing Ca^2+^ ATPase activities in STZ-induced diabetic rats (McAnuff et al., 2002, 2005). The increased action of the ATPases has direct effect on insulin, which plays major role in blood glucose regulation. Interestingly, oral administration of diosgenin (10–60 mg/kg bw) for 2 weeks decreased FBG (58%), elevated plasma insulin levels and tissue hexokinase activity with subsequent attenuation of oxidative stress in STZ-induced diabetic rats (Pari et al., 2012; Sangeetha et al., 2013; Saravanan et al., 2014). In another study, dietary inclusion of diosgenin (0.5 or 2%) for 4 weeks improved glucose tolerance ability as well as insulin sensitivity in high-fat diet-fed KK-Ay/Ta Jcl obese diabetic mice (Uemura et al., 2010).

In coherence with this finding, Naidu et al. (2015) have reported a 62.6% FBG reduction and amelioration of insulin resistance and hyperlipidemia after 30 day administration of diosgenin (60 mg/kg bw) in the same animal model. To further support this, diosgenin (10 mg/kg bw) treatment showed 70% reduction of FBG, improved antioxidant status and insulin levels in STZ-induced diabetic rats (Kalailingam et al., 2014). The higher hypoglycemic action of diosgenin was previously attributed to the reduction of serum levels of cytokines, and adipokines as well as increased PPARγ levels, implying the insulin-sensitizing potential of diosgenin in diabetic condition (Tharaheswari et al., 2014). In another study, oral treatment of diosgenin (40 mg/kg bw) for 7 weeks mitigated vascular dysfunction in STZ-induced diabetic rats (Roghani-Dehkordi et al., 2015). More recently, consumption of diosgenin (40 mg/kg bw) for 45 days decreased FBG (55%) and attenuated hyperlipidemia via inhibition of HMG-CoA reductase activity in STZ-induced diabetic rats (Hao et al., 2015).

Treatment of diosgenin (10–40 mg/kg bw) for 4 or 7 weeks demonstrated antihyperglycemic, antihyperlipidemic, cardioprotective and reno-protective potential in STZ-induced diabetic rats (Golshahi and Roghani-Dehkordi, 2016; Kanchan et al., 2016). However, Sato et al. (2014) have shown a weak reduction of FBG upon diosgenin (3 mg/kg bw) 24 h post-administration in STZ-induced diabetic rats, which may be apparently attributed to the short study period and lower dosage used.

#### Toxicity

Despite the fact that the detail toxicity studies of diosgenin has not been well documented, the oral LD_50_ was reported to be >8,000 mg/kg bw in rats (Ryndina et al., 1977). Furthermore, available toxicity studies on some animal models have shown that diosgenin (3.5% w/w) was safe and did not cause any toxicity in the treated animals (Raju and Rao, 2011).

#### Recommendations

Based on the results of the above-mentioned studies, diosgenin could be regarded as a potential hypoglycemic agent although clinical studies are required to fully confirm its hypoglycemic potential. Regardless of the few data available, diosgenin was observed to reduce FBG by >50% in several diabetic animal models and ameliorated diabetes-associated complications at non-toxic dosages (Pari et al., 2012; Sangeetha et al., 2013; Kalailingam et al., 2014; Saravanan et al., 2014; Hao et al., 2015; Naidu et al., 2015). Additionally, the potent attenuation of insulin resistance and hyperlipidemia at a concentration <10 μM is quite promising (Uemura et al., 2010; Liu et al., 2012; Sangeetha et al., 2013). Furthermore, despite few data regarding the safety issues associated with diosgenin consumption, the less toxic effect reported (LD_50_: >8,000 mg/kg bw) associated with diosgenin is of a great interest.

### Eugenol

#### *In vitro* studies

Eugenol is an active ingredient of cloves and other spices such as basil (*Ocimum basilicum* L.) and cinnamon. has diverse pharmacological potential such as hypoglycemic action. Eugenol (2.5–12.5 mM) demonstrated inhibitory actions on α-glucosidase (IC_50_: 326.1 μM) activity and advanced glycation end products (IC_50_: 10 μM) formation (Singh et al., 2016). Additionally, Mnafgui et al. (2013) highlighted that eugenol (10–100 μM) inhibited pancreatic α-amylase (IC_50_: 62.53 mg/ml) and lipase (IC_50_: 72.34 mg/ml) as well as angiotensin converting enzyme (ACE) activities (IC_50_: 130.67 mg/ml). However, despite higher IC_50_ values exhibited by the eugenol, the data signified the eugenol potential in ameliorating post-prandial hyperglycemia and diabetes-related oxidative damage and hypertension. Previously, eugenol (5–20 μM) was reported to prevent hyperglycemia in SHSY5Y cells (Prasad et al., 2015). Furthermore, eugenol (10–100 μM) stimulated muscle glucose uptake via increased GLUT4 and PI3K genes expression in L6 myotubes (Prabhakar and Doble, 2011).

#### *In vivo* studies

Dietary supplementation of eugenol (200 mg/kg bw) for 2 weeks attenuated nerve and vascular dysfunction with no significant reduction of FBG in STZ-induced diabetic rats (Nangle et al., 2006). However, Mnafgui et al. (2013) have shown 62.5% reduction of FBG with potent antioxidant potential when eugenol (80 mg/kg bw) was administered orally for 30 days in alloxan-induced diabetic rats. Srinivasan et al. (2014) have reported that eugenol (2.5–10 mg/kg bw) treatment for the same study period demonstrated antihyperglycemic and antioxidant potential in STZ-induced diabetic rats. The highest reduction of FBG was about 70.6% with improved activities of key enzymes (hexokinase, pyruvate kinase, glucose-6-phosphatedehydrogenase, glucose-6-phosphatase, fructose-1,6-bisphosphatase) related to carbohydrate metabolism (Srinivasan et al., 2014).

In another study, oral administration of eugenol at 20 and 40 mg/kg bw for 15 weeks reduced FBG by 20 and 28.6%, respectively in high fat-fed C57BL/6J mice (Jeong et al., 2014). Furthermore, oral administration of eugenol (10 mg/kg bw) for 5 days or 6 weeks showed maximum reduction of FBG by 38% and improved the *in vivo* antioxidant status of STZ-induced diabetic rats (Prasad et al., 2015; Singh et al., 2016). This variation could be linked to the different animal models used. On the other hand, Rauscher et al. (2001) have reported that intraperitoneal treatment of isoeugenol (10 mg/kg bw) for 2 weeks did not show any antihyperglycemic effect in STZ-induced diabetic rats. Additionally, a moderate antioxidant potential was reported in the treated animals, indicating weak hypoglycemic potential (Rauscher et al., 2001).

#### Toxicity

The LD_50_ of eugenol administered orally to rats was >1,000 mg/kg (Sober et al., 1950; Taylor et al., 1964; Hagan et al., 1965). However, LaVoie et al. (1986) reported a lower LD_50_ of 11 mg/kg bw after intratracheal instillation in rats. Similarly, the toxic effects manifested include lung congestion with interstitial hemorrhages, acute emphysema, and acute pulmonary edema. Recently, treatment of eugenol (0.06 μM) showed genotoxicity and cytotoxicity on dental pulp fibroblasts (Escobar-García et al., 2016). Furthermore, eugenol (3 mmol/l) induced oral mucosal fibroblasts within 2 h post-administration period (Jeng et al., 1994).

#### Recommendations

Based on the above studies the potential of eugenol as hypoglycemic agent is not consistent and thus, need further extensive studies to establish the potency of eugenol hypoglycemic action. However, some studies highlighted >60% FBG reduction at non-toxic dosages (<1,000 mg/kg bw) and attenuation of diabetes-induced complications which are quite encouraging (Mnafgui et al., 2013; Srinivasan et al., 2014; Prasad et al., 2015). Therefore, according to the current literature, the above-mentioned studies have shown the potential of eugenol as adjuvant in the diabetes management.

### Galactomannan

#### *In vitro* studies

Galactomannan is a heterogeneous water-soluble polysaccharide from fenugreek with a structural similarity to standard hypoglycemic drug, acarbose. Galactomannan (0.1 and 0.5% w/w) was reported to reduce intestinal glucose uptake in isolated intestine of lean and obese rats and thus improve glycemia (Srichamroen et al., 2009). Furthermore, galactomannan enhanced glucose uptake (51.9%) in isolated hemidiaphragm of treated alloxanized rats (Anwar et al., 2009). Kashef et al. (2008) have shown that galactomannan (200 mg/ml) inhibited the α-amylase activity. This imply that galactomannan could be beneficial in amelioration of post-prandial hyperglycemia in diabetes.

#### *In vivo* studies

Dietary inclusion of galactomannan (2.5 and 5% w/w) attenuated post-prandial hyperglycemia, hyperlipidemia and abdominal fat deposit in high sucrose-fed rats (Srichamroen et al., 2008). Oral administration of galactomannan to STZ-induced diabetic rats inhibited maltase, lactase and sucrase activities in the small intestine of treated rats (Hamden et al., 2010). These studies support the *in vitro* data and further confirm the amelioration of post-prandial hyperglycemia by the galactomannan. In another study, oral administration of galactomannan (250–500 mg/kg bw) for 3 weeks reduced FBG (59.4%) and improved serum insulin levels in alloxan induced diabetic rats (Al-Fartosy, 2015). However, a reduction of about 40% on FBG level and improved antioxidant potential were reported upon 2 h post-administration of galactomannan (500 mg/kg bw) in the same animal model (Kamble and Bodhankar, 2013; Kamble et al., 2013). Kandhare et al. (2015) have reported that chronic consumption of galactomannan (60 and 100 mg/kg bw) for 12 weeks ameliorated hyperglycemia (50%) and insulin resistance in C57BL/6 mice.

#### Toxicity

Galactomannan was reported to be safe up to 8 g/kg with no deleterious effects after 3 days post-administration period (Anwar et al., 2009; Al-Fartosy, 2015). This was similarly reported even after repeated doses for 90 days (Deshpande et al., 2016a). To further support the galactomannan safety, oral administration during gestation induced no significant maternal and embryo-fetal toxicity up to 1,000 mg/kg bw in rats (Deshpande et al., 2016b).

#### Recommendations

Studies above have shown that little information are available regarding the hypoglycemic potential of galactomannan and thus strenuous to make logical conclusion. However, our observations showed that some studies used galactomannan at high dosages (500 mg/kg bw) or concentrations (200 mg/ml) in addition to being a high molecular weight molecule, signifying weak hypoglycemic action. Therefore, more detail studies are required to fully evaluate the hypoglycemic action of galactomannan both in humans and experimental animal models.

### Gingerols and gingerol-related compounds

#### *In vitro* studies

##### Gingerol

Gingerol ([6]-gingerol) and gingerol-related derivatives (shogaol, paradol and zingerol) are the prominent ingredients of ginger and other members of Zingiberaceae.

Li and co-authors have reported that gingerols (50–150 μM) enhanced glucose uptake in L6 myotubes and muscle C2C12 cells, attributed to an increased surface availability of GLUT4 protein and by activation of AMPK in the cells (Li Y. et al., 2012; Li et al., 2013; Son et al., 2015). Available studies have shown that diabetes leads to an increase accumulation of β-amyloid, a major component of senile plaques, leading to β-cell dysfunction and failure (Maher and Schubert, 2009; Takeda et al., 2011; Luo et al., 2016). Interestingly, [6]-gingerol (2.5–20 μM) attenuated β-amyloid-induced oxidative cell death in SH-SY5Y neuroblastoma cells (Lee C. et al., 2011).

Furthermore, in a number of previous studies, [6]-gingerol was shown to play a beneficial role in reducing lipid accumulation in 3T3 cells via downregulating PPARγ and decreasing Akt/GSK3β pathway (Isa et al., 2008; Tzeng and Liu, 2013; Tzeng et al., 2014; Choi et al., 2016; Suk et al., 2016). Reducing lipid accumulation may delay the onset and progression of insulin resistance in diabetes. [6]-Gingerol (10 μM) was also reported to prevent diabetes-induced diastolic dysfunction in isolated murine ventricular myocardia (Namekata et al., 2013). In our recent study, [6]-gingerol (30–240 μg/ml) inhibited α-amylase (IC_50_: 81.8 μM) and α-glucosidase (IC_50_: 21.6 μM) actions, signifying its potential in ameliorating post-prandial hyperglycemia (Mohammed et al., 2017).

##### [6]-shogaol

[6]-Shogaol (25 μM) inhibited the TNF-α mediated downregulation of adiponectin expression in 3T3-L1 adipocytes via inhibition of c-Jun-NH_2_-terminal kinase action (Isa et al., 2008). This prevents increased production of pro-inflammatory mediators and oxidative stress markers. Wei et al. (2017) have shown that 6-shogaol (100 μM) promoted glucose utilization via AMPK phosphorylation in 3T3-L1 adipocytes and C2C12 myotubes. [6]-Shogaol (30–240 μg/ml) showed weak α-amylase (IC_50_: 443.2 μM) and α-glucosidase (IC_50_: 326.1 μM) inhibition via non-competitive mode of inhibition (Mohammed et al., 2017).

##### [6]-paradol

It has been reported that [6]-paradol (100 μM) stimulated glucose utilization via AMPK phosphorylation in 3T3-L1 adipocytes and C2C12 myotubes, which apparently improved insulin sensitivity of the target tissues (Wei et al., 2017). More recently, [6]-paradol (30–240 μg/ml) exhibited weak inhibitory actions toward α-amylase (IC_50_: 664.6 μM) and α-glucosidase (IC_50_: 243.3 μM) actions (Mohammed et al., 2017).

### *In vivo* studies

#### [6]-gingerol

Singh et al. (2009) reported that oral treatment of [6]-gingerol (100 mg/kg bw) for 12 days reduced FBG (57.1%) in db/db mice with potent antihyperlipidemic and antioxidant actions. Oral consumption of [6]-gingerol (75 mg/kg bw) for 3 weeks reduced FBG (42%) via upregulation of GLUT4, IRS-1, IRS-2, PI3K, AKT, PPARα pathways in sodium arsenate hyperglycemic mice (Chakraborty et al., 2012). This supports the previous *in vitro* studies that showed the [6]-gingerol potential to increase GLUT4 protein availability and activate AMPK (Li Y. et al., 2012; Li et al., 2013; Son et al., 2015). In addition, modulation of enzymes activities involved in gluconeogenesis and glycogenolysis was also proposed as possible mechanism involved in the hypoglycemic effect of [6]-gingerol (Son et al., 2015).

Intraperitoneal treatment of [6]-gingerol (3 or 75 mg/kg bw) for 8 weeks demonstrated antihyperglycemic (10%), cardioprotective potential, improved post-prandial glucose utilization and insulin sensitivity in STZ-induced diabetic rats (Shao et al., 2016). Similarly, Sampath et al. (2016, 2017) have shown maximum FBG (50%) reduction and potent aldose reductase inhibition upon intraperitoneal administration of [6]-gingerol (25 and 75 mg/kg bw) three times per week for 16 weeks to C57BL/6J hyperlipidemic mice. The oral treatment showed higher reduction of FBG relative to the study period compared to the intraperitoneal injection.

#### [6]-paradol

Oral administration of [6]-paradol (33.75 mg/kg bw) for 8 weeks decreased FBG (37.6%) in high-fat diet-fed mice (Wei et al., 2017).

#### Zingerone

Oral administration of zingerone (10 mg/kg bw) for 4 weeks reduced FBG (64.1%), improved the levels of hematological parameters and attenuated dyslipidemia in STZ-induced diabetic rats (Jothi et al., 2016a,b).

#### Toxicity

Fewer data are available regarding the potential toxicity associated with the intake of [6]-gingerol and its derivatives. Consumption of [6]-gingerol (20–80 μM) induced genotoxicity, lysosomal and mitochondrial damage in human hepatoma G2 (HepG2) cells (Yang et al., 2010). However, consumption of either [6]-gingerol or [6]-shogaol (2,000 mg) for 4 days was reported not to cause any potential toxicity in human subjects and are well tolerated (Zick et al., 2008). On the other hand, the LD_50_ of zingerone was reported to be 1,000 mg/kg bw (Rao et al., 2009).

#### Recommendations

From the above-mentioned studies, it is obvious that information regarding the hypoglycemic potential and toxicity of [6]-gingerol and its derivatives are scanty and that makes the overall comment inconclusive. Moreover, according to the data, [6]-gingerol showed higher hypoglycemic potential compared to its derivatives (Singh et al., 2009; Namekata et al., 2013; Sampath et al., 2016; Mohammed et al., 2017). Therefore, more studies are required on [6]-gingerol and notwithstanding, these compounds could be regarded as hypoglycemic adjuvant as reported in the above-mentioned studies.

### 4-hydroxyisoleucine

#### *In vitro* studies

4-Hydroxyisoleucine (4-OH-Ile) is an active ingredient of fenugreek and most of the hypoglycemic effect of fenugreek are attributed to 4-OH-Ile. Studies available have reported the beneficial effect of 4-OH-Ile in the control of diabetes and its associated complications. Jaiswal et al. (2012) have reported that 4-OH-Ile (5–25 μM) stimulated glucose uptake in L6-GLUT4 *myc* myotubes, which has been recently confirmed by Korthikunta et al. (2015). In another study, 4-OH-Ile (10 μM) ameliorated insulin resistance in L6 myotubes (Rawat et al., 2014). Moreover, using the same model, 4-OH-Ile (5–25 μM) was shown to ameliorate insulin resistance and demonstrated potent anti-inflammatory action (Maurya et al., 2014). In HepG2 cells, 4-OH-Ile (100 ng/ml) promoted insulin signaling and the expression of glycogenic enzymes and GLUT2 (Naicker et al., 2016). Previously, 4-OH-Ile (100–1,000 μM) stimulated insulin release in isolated rat pancreas (Sauvaire et al., 1998; Broca et al., 2000; Wang et al., 2002). Therefore, it is clear that 4-OH-Ile showed potential to ameliorates insulin resistance. The possible mechanisms involve increased Akt phosphorylation and reduced activation of Jun N-terminal kinase (JNK)1/2, extracellular signal-regulated kinase (ERK)1/2, p38 mitogen-activated protein kinase (MAPK), and nuclear factor (NF)-κB (Avalos-Soriano et al., 2016).

#### *In vivo* studies

Haeri et al. (2012) have reported that oral administration of 4-OH-Ile (50 mg/kg bw) for 4 weeks to STZ-induced diabetic rats reduced FBG (41%) with potent hypolipidemic and insulinotropic actions. In another study, a reduction of 34% on FBG was reported in STZ-induced diabetic rats upon treatment of 4-OH-Ile (50 mg/kg bw) for 8 weeks (Narender et al., 2006; Haeri et al., 2009). Intraperitoneal administration of 4-OH-Ile (18–50 mg/kg bw) for 15 min or 5 days showed insulinotropic action with no effect on blood glucose levels in Zucker diabetic fa/fa or STZ-induced diabetic rats (Broca et al., 1999, 2004). This supports the insulinotropic potential of 4-OH-Ile and is in line with *in vitro* data (Sauvaire et al., 1998; Broca et al., 2000; Wang et al., 2002). In C57BL/KsJ-db/db mice, oral treatment of 4-OH-Ile (50 mg/kg bw/ 10 days) lowered FBG by 55.4% (Singh et al., 2010). Consumption of 4-OH-Ile (40 mg/kg bw) for 7 weeks showed antihyperglycemic action and a potent pancreatic β-cell regeneration in alloxan-induced diabetic mice (Shah et al., 2009).

#### Clinical trials

Nuttall et al. (2008) have reported that ingestion of 4-OH-Ile (1 mmol/kg lean body mass) reduced blood glucose levels and improved utilization compared to the untreated non-diabetic subjects after 4 h post-administration period.

#### Toxicity

Oral LD_50_ value of 4-OH-Ile was reported to be >5 g/kg bw indicating that consumption of 4-OH-Ile has no potential toxic effect (Shah et al., 2009).

#### Recommendations

Our candid opinion here is that 4-OH-Ile did not show a significant hypoglycemic action despite long administration period. However, based on the available data, 4-OH-Ile is insulinotropic and could be used in combination with other drugs to attenuate diabetes-induced oxidative damage, and hence regarded as an adjuvant. Most importantly, detail toxicological studies are required to evaluate the safety of 4-OH-Ile both in humans and experimental animals.

### Piperine

#### *In vitro* studies

Piperine is the major alkaloid responsible for the pungency of black pepper (*Piper nigrum* L.). Kumar S. et al. (2013) have reported that piperine showed weak inhibition toward α-lipase (IC_50_: 2,490 μg/ml), α-glucosidase (IC_50_: 2,550 μg/ml) and aldose reductase (IC_50_: 2,375 μg/ml) activities. Inhibition of the activities of these enzymes signify its potential in attenuating diabetes-associated complications.

#### *In vivo* studies

Kharbanda et al. (2016) have reported that piperine (36 mg/kg bw) isolated from black pepper demonstrated antihyperglycemia in STZ-induced diabetic rats by acting as PPAR-γ agonists. Interestingly, Atal et al. (2016) have shown that co-administration of piperine (10 mg/kg bw) with metformin for 4 weeks reduced FBG (40%) compared to metformin alone (19%) in STZ-induced diabetic mice, indicating synergistic effect between the two drugs. Furthermore, weak reduction on FBG was observed upon administration of piperine (10–50 mg/kg bw) for the same study period and model (Rauscher et al., 2000; Kharbanda et al., 2016). However, the levels of serum insulin, lipid profiles and antioxidant enzymes were significantly improved. Oral administration of piperine (20 or 40 mg/kg bw) for 11 weeks ameliorated hyperglycemia (40%) and oxidative damage in STZ-induced diabetic rats (Arcaro et al., 2014). On the other hand, several studies have reported the beneficial effect of piperine in reducing hyperglycemia and attenuating oxidative stress in high-fat diet rats (Vijayakumar et al., 2004; Shah et al., 2010; Bao et al., 2012; BrahmaNaidu et al., 2014).

#### Toxicity

The safety aspect of piperine has been controversial. Piyachaturawat et al. (1983) have reported that the LD_50_ values of piperine via different route are in the order intravenous (15.1 mg/kg bw) < intraperitoneal (43 mg/kg bw) < subcutaneous (200 mg/kg bw) < intragastric (330 mg/kg bw) < intramuscular (400 mg/kg bw). The authors further showed that almost all the animals that received a lethal dose (>LD_50_) died from respiratory complications in <20 min. However, during sub-chronic study, the death occurred within l−3 days after post-administration period. Some of the histopathologic alterations observed include severe hemorrhagic necrosis and edema in GIT, urinary bladder and adrenal glands (Piyachaturawat et al., 1983). Additionally, its toxic effect has been attributed to its structural similarity with some known carcinogens such as safrole, estragole, and methyleugenol (Ames, 1983). On the other hand, consumption of piperine orally (170 mg/kg bw) or intraperitoneally (85 mg/kg bw) did not cause any adverse consequences in rats, with 3% excreted as piperine in the feces (Bhat and Chandrasekhara, 1986).

#### Recommendations

The information derived from the above-mentioned studies revealed that piperine is a weak hypoglycemic agent despite longer administration period. None of the studies have shown up to 50% reduction on blood glucose levels. The higher IC_50_ value depicted toward α-glucosidase and aldose reductase inhibitions indicated weak hypoglycemic action as well. Another major concern is the contradiction on the safety issues regarding pure piperine consumption. However, piperine could be regarded as food adjuvant in the management of diabetes based on the potent antioxidant action observed in the above-mentioned studies. Additionally, the use piperine as a naturally-based bio-enhancers to some drugs is receiving much attention and yielding fruitful results (Moorthi and Kathiresan, 2013; Arcaro et al., 2014).

### Thymoquinone

#### *In vitro* studies

Thymoquinone (TQ) is the main pharmacologically active ingredient of black cumin seeds (*Nigella sativa* L.), with proven hypoglycemic potential. TQ (2.5 μM) promoted glucose-stimulated insulin secretion and attenuated oxidative damages induced by protease inhibitors in rat pancreatic β-cells Chandra et al. (2009). Previously, TQ (10–50 μM) demonstrated potent antiglycation (IC_50_: 7.2 μM) potential (Losso et al., 2011; Anwar et al., 2014; Khan et al., 2014). Furthermore, TQ (3 mg/kg) reduced diabetes-induced elevated levels of macrophage-derived inflammatory mediators such as TNF-α, nitrite and IL-1β in isolated STZ-induced diabetic rat model peritoneal macrophages (El-Mahmoudy et al., 2005a). More recently, TQ (0–5 μM) improved insulin secretion from pancreatic β-cells in INS-1 cells (Gray et al., 2016).

#### *In vivo* studies

Thymoquinone (0.5–6 mg/kg bw) administered intraperitoneally showed hypoglycemic (39.7% reduction) potential in non-diabetic rats (Hawsawi et al., 2001). Oral administration of TQ (50 mg/kg bw) for 12 weeks reduced FBG (37%), stimulated insulin release and improved histopathological changes in sciatic nerves of the STZ-induced diabetic rats (Kanter, 2008, 2009). However, a reduction of 45% on FBG was reported upon oral gavage of TQ (40 mg/kg bw) for 3 weeks in the same animal models (Bashandy et al., 2015). Interestingly, similar reduction of FBG was observed after 2 h post-TQ (60 mg/kg bw) administration period in STZ-nicotinamide-induced diabetic rats (El-Ameen et al., 2015).

In some studies, TQ (50 mg/kg bw) treatment for 3 or 4 weeks reduced FBG by 63% compared to 76% in insulin treated rats in the same animal models (Fararh et al., 2005, 2010). In addition, the activities of hepatic gluconeogenic enzymes were decreased (Fararh et al., 2010). Moreover, oral administration of TQ (20–80 mg/kg bw) for 45 days reduced FBG (61%), improved glucose tolerance, serum insulin levels and antioxidant status in STZ-nicotinamide-induced diabetic rats (Pari and Sankaranarayanan, 2009; Roghani and Baluchnejadmojarad, 2012). Additionally, improved activities of hexokinase, glucose 6-phosphate dehydrogenase, glucose 6-phosphatase and fructose 1, 6-bisphosphatase were observed as well.

To further support this, administration of TQ (20–80 mg/kg bw) for 12 weeks reduced FBG (>70%) STZ-nicotinamide-induced diabetic rats (Fouad and Alwadani, 2015). Oral TQ (80 mg/kg bw) consumption ameliorated diabetes-induced pancreatic oxidative damages with subsequent improvement of antioxidant status in the same animal model (Sankaranarayanan and Pari, 2011a,b). Furthermore, considerable data are available showing TQ (3–50 mg/kg bw) potential in attenuating diabetes-induced oxidative damages via increased expression of antioxidant enzymes in STZ-induced diabetic or hyperlipidemic rats (Abdelmeguid et al., 2010; Mehrdad and Tourandokht, 2012; Ahmad and Beg, 2013; Al Wafai, 2013; Elmansy and Almasry, 2013; Hafez, 2013; Ashour, 2015; Bashandy et al., 2015; Desai et al., 2015; Al-Trad et al., 2016; Saheb et al., 2016).

Salehi et al. (2012) have reported that TQ treatment (2.5 and 5 mg/kg bw) for 5 weeks improved the spatial memory in STZ-induced diabetic rats via attenuation of lipid peroxidation. Furthermore, oral gavage of TQ (10 mg/kg bw) for 2 weeks reduced FBG (66%) and improved the antioxidant status of STZ-induced diabetic rats (Hamdy and Taha, 2009). El-Mahmoudy and colleagues have reported similar reduction of FBG upon oral consumption of TQ (3 mg/kg bw) for 30 days in LETO-STZ-induced diabetic rats (El-Mahmoudy et al., 2005b). Supplementation of TQ (20 mg/kg bw) during pregnancy and lactation periods to STZ-induced gestational diabetic rats induced FBG (20%) and pro-inflammatory cytokines levels (IL-1b, IL-6, IL-2, and TNF-α) in the offspring (Badr et al., 2011, 2013). Surprisingly, TQ administered intraperitoneally at 3 and 5 mg/kg bw for 8 weeks reduced FBG by 68 and 66%, respectively which could be attributed to the longer period of administration (Sangi et al., 2015).

#### Toxicity

Tremendous efforts were made to assess the toxicological properties of TQ using various *in vitro* and *in vivo* models (El-Dakhakhny, 1965; Badary et al., 1998; Mansour et al., 2001; Al-Ali et al., 2008; Khader et al., 2009; Qadri et al., 2009; Abukhader, 2012). The LD_50_ of TQ in rats via oral and intraperitoneal administration were 794.3 and 57.5 mg/kg, when in mice the values were 870.9 and 104.7 mg/kg through oral and intraperitoneal route, respectively (Al-Ali et al., 2008). Previously, Badary et al. (1998) have shown that the acute LD_50_ value in mice was 2.4 g/kg bw via oral ingestion of TQ. This indicates the relatively low toxicity of TQ since the LD_50_ values were >10 and >100 times higher than the therapeutic dosages for TQ via intraperitoneal and oral routes, respectively. However, few signs of the toxicity such as hypoactivity and difficulty in respiration were observed after acute oral administration of TQ in rats (Badary et al., 1998).

Moreover, sub-chronic administration of TQ (35 and 50 mg/kg bw) induced disruption on embryonic development during the second trimester of rat pregnancy (Abukhader, 2012). Conversely, TQ (30–90 mg/kg bw/day) administration for 3 months caused no mortality or sign of toxicity in mice (Badary et al., 1998). Interestingly, Al-Amri and Bamosa (2009) have reported that oral ingestion of TQ for 3 weeks did not show any potential toxicity and was well tolerated up to dose of 2,600 mg/kg bw in human subjects. However, according to the authors TQ administration showed no therapeutic potential up to the maximum dosage used (Al-Amri and Bamosa, 2009).

#### Recommendations

Based on the above-mentioned studies, TQ possessed blood glucose lowering potential and could be used to attenuate diabetes-induced complications despite lack of relevant clinical trials. Our rationale is that TQ demonstrated hypoglycemic potential at 3–50 mg/kg bw in animal models (Hamdy and Taha, 2009; Pari and Sankaranarayanan, 2009; Roghani and Baluchnejadmojarad, 2012) and depicted IC_50_ value <10 μM at the concentrations (10–50 μM) in addition to stimulating insulin release at 2.5 μM (Chandra et al., 2009). Moreover, most of the studies have reported more than 50% reduction on blood glucose levels and potent antioxidant actions (Fararh et al., 2005, 2010; Hamdy and Taha, 2009; Pari and Sankaranarayanan, 2009; Roghani and Baluchnejadmojarad, 2012; Fouad and Alwadani, 2015; Sangi et al., 2015). However, lack of detail hypoglycemic and toxicity studies in human subjects are the major concerns.

### Trigonelline

#### *In vitro* studies

Trigonelline is a spice-derived alkaloid from fenugreek and possesses tremendous therapeutic potential including hypoglycemic potential. Trigonelline (0.33 and 3.3 mg/ml) inhibited glucose uptake (IC_50_: 19 mM) in isolated intestinal rabbits (Al-Habori et al., 2001). More recently, Ilavenil et al. (2014) have shown that trigonelline (75 or 100 μM) attenuated adipocyte differentiation and subsequent hyperlipidemia in 3T3-L1 cells.

#### *In vivo* studies

Oral administration of trigonelline (50–100 mg/kg bw) for 4 weeks showed maximum FBG reduction of 27%, attenuated TNF-α levels and improved insulin levels in neonatal STZ-induced diabetic rats (Ghule et al., 2012). In addition, glomerular filtration rate, activities of antioxidant enzyme and membrane bound enzymes were improved in treated animals. Subsequently, oral consumption at 10 mg/kg bw for 4 weeks demonstrated antihyperglycemia, antihyperlipidemic and antioxidant potential in alloxan-induced diabetic rabbits (Monago and Nwodo, 2010; Al-Khateeb et al., 2012). The highest reduction of FBG was 74.5% compared to 61.1% for Chlorpropamide (Monago and Nwodo, 2010). More recently, supplementation of trigonelline (150 mg/kg bw) for 30 days reduced FBG (50%), hyperlipidemia and diabetes-induced oxidative damages in high-fat diet-fed low-dose STZ-induced diabetic rats model (Subramanian and Prasath, 2014a,b).

Furthermore, Shah and colleagues have reported similar reduction of FBG (>50%) after 24 h treatment in alloxan-induced diabetic rats (Shah et al., 2006). Additionally, repeated oral administration of trigonelline (75 mg/kg bw) for 7 days have shown about 57% reduction on FBG and improved the histology of pancreas of treated rats (Shah et al., 2006). Interestingly, a potent antioxidant potential of trigonelline (10 mg/kg bw) was later documented in alloxan-induced diabetic rats (Hamadi, 2012). Trigonelline (25–100 mg/kg bw) exhibited maximum FBG reduction of 16% after acute (24 h) administration period in nicotinamide STZ-induced diabetic rats (Kamble and Bodhankar, 2014). However, after trigonelline (50 mg/kg bw) treatment for 8 weeks the FBG reduction was lower (48%) as compared to trigonelline treated and sitagliptin (5 mg/kg bw) combination (63%) when same animal models were used (Kamble and Bodhankar, 2013,b).

Dietary inclusion of trigonelline (0.056%) for 43 days demonstrated weak hypoglycemic action (<10% FBG reduction) and potent antioxidant potential in Goto-Kakizaki type 2 diabetes rats (Yoshinari et al., 2009, 2013; Yoshinari and Igarashi, 2010). In another study, supplementation of trigonelline (40 mg/kg bw) for 48 weeks reduced FBG (75%), ameliorated insulin resistance and peripheral diabetic neuropathy in high-fat diet-fed STZ-induced diabetic rats (Zhou and Zhou, 2012). Furthermore, the same authors have reported the FBG reduction of about 70% after 4-week post-administration period at the same dose and in the same animal model (Zhou et al., 2011). However, a weak FBG reduction (38%) of trigonelline was later reported upon treatment for either 2 or 4 weeks at the same dose and animal models, attributed to the short study period (Tharaheswari et al., 2014, 2015). Conversely, Hamden et al. (2013a) have reported 50% FBG reduction after oral administration of trigonelline (100 mg/kg bw) for 30 days in alloxan-induced diabetic rats. Moreover, the authors showed that trigonelline treatment significantly inhibited the activities of dipeptidyl peptidase-IV, α-glucosidase and angiotensin converting enzyme (Hamden et al., 2013a,b). Interestingly, trigonelline (50 mg/kg bw) treatment orally for 4 weeks reduced FBG (81%) in nicotinamide-STZ-induced diabetic rats (Folwarczna et al., 2016). More recently, similar finding was also noticed after 2-week post-administration of trigonelline (50–100 mg/kg bw) in fructose-induced insulin resistance (Ramadan et al., 2016).

#### Clinical trials

Ingestion of trigonelline (500 mg) reduced blood glucose by about 7% and improved glucose tolerance after 15 min post-treatment period in overweight subjects (Van Dijk et al., 2009). The hypoglycemic action was found not to be dependent on the incretin hormones glucagon-like peptide 1 (GLP-1) or glucose-dependent insulinotropic peptide (Olthof et al., 2011).

#### Toxicity

Aswar et al. (2009) have shown that oral consumption of trigonelline was safe up to 5,000 mg/kg bw with no noticeable abnormal behavior in rats.

#### Recommendations

As per data from the above studies, trigonelline seems to be among the promising hypoglycemic agents despite few studies which showed weak or no significant hypoglycemic potential. Longer administration period such as 4 weeks at 10–100 mg/kg bw showed potent reduction (>50%) of FBG in diabetic rats (Shah et al., 2006; Monago and Nwodo, 2010; Al-Khateeb et al., 2012; Zhou et al., 2011; Hamden et al., 2013a; Subramanian and Prasath, 2014a,b; Folwarczna et al., 2016). Additionally, a potent amelioration of diabetes-induced complications was observed even in those studies that showed weak hypoglycemic potential. The weak blood glucose lowering potential in humans could be attributed to the shorter study period (Van Dijk et al., 2009). Although few data are available regarding trigonelline toxicity, its consumption did not show any potential toxic effect in rats (Aswar et al., 2009).

#### Bioavailability of spice-derived ingredients

Conventionally, poor bioavailability is considered as a major factor linked to the lower therapeutic efficacy of the orally consumed SDBI. Therefore, improving bioavailability of the ingredients is a promising approach in enhancing their disease preventing efficacy in humans. The oral bioavailability of SDBI entails the portion of the ingested ingredient that get in to the blood circulation in its active form. Because, only bioavailable portion will be absorbed and distributed across the tissues and organs that eventually exert its therapeutic effects. Moreover, poor solubility in gastrointestinal fluids and slow absorption rate from the GIT are the crucial factors that thwart SDBI from reaching the systemic circulation in their active forms (Yao et al., 2015).

According to our critical observation, cinnamaldehyde, curcumin, diosgenin, TQ and trigonelline are the promising hypoglycemic SDBI despite their known poor bioavailability in the physiological system. Interestingly, there has been a renewed interest in developing methods that may improve the bioavailability of the SDBI to prevent or treat human diseases such as diabetes. In this regard, we have briefly presented some fact regarding the bioavailability of these promising ingredients and the methods being used to improved their bioavailability.

#### Bioavailability of cinnamaldehyde

Cinnamaldehyde is absorbed rapidly from the gut, utilized and excreted via urine, regardless of the dosages, species and sex of the animals used. Oral consumption of cinnamaldehyde was shown to be metabolized into cinnamic acid partially in the stomach and small intestine and then completely metabolized into cinnamic acid in the liver before it enters the circulation (Chen et al., 2009). Previously, Yuan et al. (1993) and Peters and Caldwell (1994) have reported that the intravenous administration of the various dosages of cinnamaldehyde (5–25 mg/kw bw) to F344 rats decreased blood glucose 30 min after the dose administration. The disappearance of cinnamaldehyde is attributed to the rapid oxidation to cinnamic acid in blood (about 37–60%). This is because 1.7 h half-life has been considered for cinnamaldehyde release from the protein adducts (Yuan et al., 1992). Furthermore, the authors highlighted that the blood level of cinnamaldehyde after oral consumption was maintained 1 μg/ml for 24 h (Yuan et al., 1992). More recently, the elimination time of cinnamaldehyde (125–500 mg/kg bw) were 6.7 and 1.7 h for oral and intravenous administration, respectively and the oral bioavailability of about 20% in the blood (Hooth et al., 2004).

#### Improving cinnamaldehyde bioavailability

Based on the data available, improving cinnamaldehyde bioavailability focused on three major processes including use of cinnamaldehyde derivatives or metabolites, micelle, microencapsulation and nanoparticles approaches (Hooth et al., 2004; Raffai et al., 2014; Wani et al., 2014; Jo et al., 2015). However, with the exception of using cinnamaldehyde derivatives, none of the methods was so far employed regarding the hypoglycemic potential of cinnamaldehyde either *in vitro, in vivo* or in human subjects. These approaches could be used to explore the hypoglycemic potential of cinnamaldehyde and therefore, warrant for further study in this regard.

#### Bioavailability of curcumin

It is well-established that curcumin is poorly bioavailable and thus its pharmacological effects are compromised. The low plasma and tissue levels of curcumin has been attributed not only to its poor absorption but rapid hepatic metabolism and systemic elimination (Anand et al., 2007; Cui et al., 2009; Bansal et al., 2011). For instance, about 51 ng/ml of curcumin was detected in the serum after 4 h oral consumption of curcumin (12 g) in healthy human subjects (Lao et al., 2006b). However, Marczylo et al. (2009) showed a relatively higher distribution of curcumin (340 mg/kg bw) in plasma (16.1 ng/ml), urine (2.0 ng/ml), intestinal mucosa (1.4 mg/g), liver (3,671.8 ng/g), kidney (206.8 ng/g), and heart (807.6 ng/g) after 2 h post-oral treatment. Previously, oral ingestion of curcumin (400 mg) showed about <20 μg/tissue levels in the kidney or liver, when no curcumin or trace amount was found in the urine in rats after 24 h post-administration period (Ravindranath and Chandrasekhara, 1982).

Furthermore, about 60–67% of curcumin (10–400 mg/kg bw/ 12 days) was absorbed and maintained at relatively constant amount in the circulation independent of the dose administered (Ravindranath and Chandrasekhara, 1982). In another study, administration of curcumin (0.1 g/kg bw) intraperitoneally showed tissue distribution of 177.04, 26.06, 26.90, 7.51, and 0.41 μg/g in the intestines, spleen, liver, kidneys and brain, respectively after 1 h treatment in mice (Pan et al., 1999). Regarding the curcumin metabolism, curcumin undergoes bioreduction to dihydrocurcumin and tetrahydrocucurmin which are then converted to either glucuronide or sulfate conjugates in the body system (Garcea et al., 2004).

#### Improving curcumin bioavailability

In summary, the above-mentioned studies demonstrated the poor bioavailability of curcumin. Interestingly, tremendous efforts are introduced to alternatively increases the bioavailability, prolonged circulation, better permeability, and resistance to metabolic reactions of curcumin. These processes include the use of everted sacs of rat intestines, use of adjuvant that interferes with glucuronidation and the use of liposomal curcumin (Suresh and Srinivasan, 2007; Shaikh et al., 2009). Others are the use of nanoparticles, curcumin phospholipid complex and the structural analogs of curcumin (Suresh and Srinivasan, 2007). For instance, the oral bioavailability of curcumin was reported to improve by 9-fold using nanoparticles approach (Shaikh et al., 2009). Moreover, the bioavailability of THC has been recently shown to be higher compared to the curcumin (Aggarwal et al., 2014).

However, with regard to improving hypoglycemic potential of curcumin, available literatures have shown that the use of curcumin derivatives and nanoparticles approaches were employed in some diabetic models. The hypoglycemic potential of the former has been addressed in the earlier section of this review (Pari and Murugan, 2005, 2007b,?; Murugan and Pari, 2006a,b; Murugan et al., 2008; Karthikesan et al., 2010a,b; Lekshmi et al., 2012a,b). For the later, Grama et al. (2013) have reported that oral administration of nano-curcumin (2 mg/kg bw) for 11 weeks reduced FBG (37%) and delayed cataract formation in STZ-induced diabetic rats. Recently, intranasal delivery of nano-micelle curcumin for 7 days was shown to significantly promote corneal epithelial/nerve healing in STZ-induced diabetic mice (Guo et al., 2016). In a randomized clinical trial, ingestion of nano-curcumin (80 mg) for 3 months reduced FBG and glycated hemoglobin by about 32 and 19%, respectively in type-2 diabetic patients (Rahimi et al., 2016).

Based on the above few studies, it is obvious that nanoparticles approach may be another option to improve the antihyperglycemic as well as hypoglycemic efficacy of curcumin when compared to the use of curcumin metabolites or derivatives as pure compounds. However, the reduction of FBG was less with curcumin nanoparticles compared to that of curcumin alone or its derivatives. Hence, further studies are required to ascertain the efficacy of curcumin nanoparticles or come up with a more improved method.

#### Bioavailability of diosgenin

The therapeutic applications of diosgenin are greatly tempered due to the poor pharmacokinetics. Cayen et al. (1979) have reported that 1 μg/ml of diosgenin was recovered from the serum of human subjects that received diosgenin (3 g/day) for 4 weeks, indicating poor absorption and bioavailability of diosgenin. Furthermore, oral bioavailability of diosgenin was highlighted to be 6% in rats and aqueous solubility was found to be 0.95 μg/ml (Okawara et al., 2010, 2013).

#### Improving diosgenin bioavailability

To improve the solubility and intestinal permeability of diosgenin, Kim et al. (2012) have shown that conjugating the hydrophilic unit, tetraethylene glycol to form diosgenin-tetraethylene glycol conjugate improved the hypoglycemic action of diosgenin. Although both the diosgenin and the conjugate treatment (10–20 mg/kg bw) for 9 weeks did not show any significant FBG reduction, the conjugate treated group showed better potential compared to the diosgenin alone (Kim et al., 2012; Okawara et al., 2013). Interestingly, some methods are available to improve the bioavailability of diosgenin, although not directly investigated in any diabetic model. The use of diosgenin and β-cyclodextrin inclusion complex, deglycosylation of diosgenin and diosgenin nanocrystals are receiving much attention in the recent years (Gao et al., 2012; Okawara et al., 2013, 2014; Liu et al., 2016). For instance, the use of diosgenin and β-cyclodextrin inclusion complexes improved the bioavailability of diosgenin by 45% in rats (Okawara et al., 2013).

#### Bioavailability of thymoquinone

Poor bioavailability in the systemic circulation has been highlighted as the major limitation for using TQ in clinic trials. Alkharfy et al. (2015) have attributed the poor bioavailability of TQ to the rapid elimination and relatively delay absorption following oral administration. Previously, Pathan et al. (2011) have detected TQ in the plasma (about 58%) for 12 h after oral administration of TQ (20 mg/kg bw) in rats. In another study, TQ was reported to accumulate greatly in the entire nuclei of kidney cells (Effenberger-Neidnicht et al., 2011).

#### Improving thymoquinone bioavailability

The consequences of TQ hydrophobicity leads to reduce amounts reaching the target which in turn increase toxicity to normal tissues. Taking into consideration many researchers have developed more aqueous-soluble TQ derivatives and encapsulate in a nanoformulations to overcome the poor bioavailability of TQ. The encapsulation increases the bioavailability, protects the TQ from prematured enzyme degradation and limits TQ diffusion to normal tissues (Schneider-Stock et al., 2014). Interestingly, this method has been widely used in various disease condition and improved actions with less toxicity were reported (Ravindran et al., 2010; Singh et al., 2013; Ong et al., 2016; Kalam et al., 2017). Unfortunately, no study is available that reported the potential of TQ-derived nanoparticle in diabetic models and hence further studies in this regard are warranted.

#### Bioavailability of trigonelline

The solubility of trigonelline was reported to be higher compared to other SDBI and thus showed moderate rate of absorption and high elimination rate in the rabbit (Zhao et al., 2003). Similarly, most of the consumed trigonelline is usually absorbed at the small intestine and not degraded by the microflora in germ-free and specific pathogen-free rats (Yuyama, 1999). Consumption of coffee (another rich-source of trigonelline) showed peak plasma concentrations of 5.5–6.5 μM after 2–3 h in human subjects (Lang et al., 2010, 2013). The authors further showed delay clearance and, hence, accumulation of trigonelline in the plasma with an average half-life of about 5 h. In addition, about 50% of the trigonelline consumed was detected in urine 0–8 h post-ingestion (Lang et al., 2010, 2013). Moreover, about 20% of the ingested trigonelline was reported to be excreted in the urine as trigonelline when approximately 9% was excreted as N'-methyl-2-pyridone-5-carboxylic acid (Yuyama and Suzuki, 1991; Yuyama and Kawano, 1996). However, about 100% of trigonelline was recovered unchanged from the urine in the administered rats (Shibata and Taguchi, 1991). Hence, further studies are needed not only to confirm the true metabolism of trigonelline but also to clear the above-mentioned controversies.

#### Improving trigonelline bioavailability

According to the literature, no study is available that highlight the method that could improve the trigonelline bioavailability. This could be attributed to the higher solubility of trigonelline compared to the other SDBI.

## Conclusion and future prospect

Data gathered in the present study have shown that SDBI hold promising hypoglycemic potential. Despite many of the ingredients showed weak hypoglycemic effects, cinnamaldehyde, curcumin, diosgenin, TQ and trigonelline have demonstrated promising hypoglycemic potential and need further scientific scrutiny to maximize their use as hypoglycemic therapies and adjuvants. Therefore, future studies on the most promising SDBI should be focused on bringing these ingredients to the forefront for the treatment of diabetes. These can be achieved via extensive clinical trials, improving the tissue bioavailability and distribution as well as detail toxicological studies. Furthermore, more detail studies are required on the combinatory effects of standard hypoglycemic drugs and the ingredients that showed adjuvant property. The idea is to evaluate whether the combined administration could attenuate advance consequences of the synthetic drugs as they showed strong amelioration of diabetes-associated complications.

## Author contributions

AM gathered all the previously published articles and drafted the manuscript in its current form under the direct guidance of MI.

### Conflict of interest statement

The authors declare that the research was conducted in the absence of any commercial or financial relationships that could be construed as a potential conflict of interest.
