# Supplementary material for: Spice-Derived Bioactive Ingredients: Potential Agents or Food Adjuvant in the Management of Diabetes Mellitus
Source: Front Pharmacol. 2018 Aug 22;9:893. doi: 10.3389/fphar.2018.00893 (PMC6113848; doi:10.3389/fphar.2018.00893)
Supplement: Supplementary file 1 [file Table_1.pdf]

Supplementary Table 1: *In vivo* studies on spice-derived ingredients

**Compounds**	**Dosages/models/ periods**	**Efficacy**	**References**
Allicin	250 mg/kg bw/day (p.o.) after 4-hour/ alloxan-induced diabetic rats	Antihyperglycemic, Improved glucose utilization	Mathew and Augusti, 1973
Alliin	200 mg/kg bw/day (p.o.) for 30 days/ alloxan-induced diabetic rats	Hepato- and cardio-protective, Antihyperglycemic, Antioxidative	Sheela and Augusti, 1992; Sheela et al., 1995; Augusti and Sheela, 1996
S-Allyl cysteine	1 g/kg bw/day (p.o.) for 4 weeks/ STZ-induced Balb/cA mice	Antihyperglycemic	Hsu et al., 2004
	150 mg/kg bw/day (p.o.) for 45 days/ STZ-induced diabetic rats	Antihyperglycemic, Antioxidative, Increases circulating thyroid hormone secretory effects	Saravanan et al., 2009; Saravanan and Ponmurugan, 2010; 2011; 2012; Saravanan et al. 2010; 2013; Saravanan and Ponmurugan, 2011
	0.5 mg/kg bw/day (p.o.) for 10 weeks/ STZ-induced diabetic mice	Antihyperglycemic, Antiinflammatory	Mong and Yin, 2012
Diallyl trisulfide	80 mg/kg bw/day (i.p.) for 3 weeks/ STZ-induced diabetic rats	Antihyperglycemic, Ameliorates insulin resistance	Liu et al., 2005; 2006
	53.5 mg/kg bw/day (p.o.) for 55 days/ Cohen-Rosenthal diabetic hypertensive rats	Antihyperglycemic	Younis et al., 2010
	40 mg/kg bw/day (i.p.) for 16 days/ STZ-induced diabetic rats	Antihyperglycemic	Kuo et al., 2013
Capsaicin	20-50 mg/kg bw/day (i.p.) for 8 weeks/ Zucker diabetic fatty (ZDF) rats	Antihyperglycemic, Immunoprotective	Gram et al., 2007

	0.015%/day (D.I.) for 3 weeks/ KK-Ay diabetic mice	Antihyperglycemic, Antihyperlipidemic, Improved insulin sensitivity	Kang et al., 2011
	0.024, 0.042%/day (D.I.) for 4 weeks/ KK-Ay diabetic mice	Moderately prevent hyperglycemia, hyperlipidemia, hyperinsulinemia	Okumura et al., 2012
	10 µg/kg bw for 20 weeks/ non-obese diabetic mouse		Nevius et al., 2012
	0.025%/day (D.I.)/ pancreatectomized diabetic rats	Antihyperglycemic, Improved insulin sensitivity	Kwon et al., 2013
	0.014, 0.1%/day (D.I.) for 12 weeks/ STZ-induced diabetic mice	Antioxidative	Hsia et al., 2016
Capsiate	0.025%/day (D.I.)/ pancreatectomized diabetic rats	Antihyperglycemic, Improved insulin sensitivity	Kwon et al., 2013
Cinnamaldehyde	5-20 mg/kg bw/day for 45 days/ STZ-induced diabetic rats	Antihyperglycemic, Antihyperlipidemic, Insulinotropic, Antioxidative	Babu et al., 2007; 2014
	40 mg/kg bw/day for 4 weeks/ high-fat diet-STZ-induced diabetic rats	Antihyperglycemic, Antihyperlipidemic, Ameliorated insulin resistance and up-regulated GLUT4 protein expression	Zhang et al., 2008a
	20 mg/kg bw/day (p.o.) for 2-4 months/ STZ-induced diabetic or C57BLKS/J db/db mice rats	Antihyperglycemic, Antihyperlipidemic, Antihypertensive, Antioxidative Improved glycolytic enzymes action	Anand et al., 2010; El-Bassossy et al., 2011; Kumar et al., 2012; Li et al., 2012a
	0.2%/day (D.I.)/ for 36 days/ in C57BL6 diabetic mice	Antihyperglycemic	Camacho et al., 2014

	143.8 μmol/kg bw/day for 4 weeks/ high-fat diet-induced rats	Antihyperglycemic, Antihyperlipidemic, Antiinflammatory, Antioxidant	Farrokhfall et al., 2014
	10-40 mg/kg bw/day for 4 weeks/ high-fat diet-STZ-induced diabetic rats	Antihyperglycemic, Decrease IL-6 and TNFα levels	Jawale et al., 2016
Curcumin	0.5%/day (D.I.) for 8 weeks/ STZ-induced diabetic rats	Antihyperlipidemic Ameliorated renal lesions	Babu and Srinivasan, 1997b; 1988
	10-80 mg/kg bw/day for 3, 4 or 8 weeks/ alloxan- or STZ-induced diabetic rats	Antihyperglycemic Antihyperlipidemic Antiglycative Antioxidative Ameliorated diabetic encephalopathy	Arun and Nalini 2002; Mahesh et al., 2004; Pari and Murugan, 2005; Sharma et al., 2006; Kuhad and Chopra, 2007; Murugan and Pari, 2007a
	0.001-0.24% (w/v)/day (D.I.) for 5, 4 or 8 weeks/ STZ-induced diabetic, high-fat diet-fed rats or KKAy diabetic or C57BL/KsJ-db/db type 2 diabetic mice	Anticataract effects Antihyperlipidemic, Antioxidative Promoted hepatic glycolysis Ameliorated diabetic retinopathy	Suryanarayana et al., 2005; Kempaiah and Srinivasan, 2006; Honda et al., 2006; Suryanarayana et al., 2007; Mrudula et al., 2007; Kowluru and Kanwar, 2007; Seo et al., 2008
	7.5 mg/kg bw/day (p.o.) for 3 weeks/ C57/BL6J diabetic rats	Antioxidant	Kanitkar et al., 2008
	60-200 mg/kg bw/day (p.o.) for 2, 3, 4, 7, 12 weeks/ STZ-induced diabetic rats	Antihyperglycemic, Ameliorates diabetic neuropathy, Antioxidant	Peeyush et al., 2009; Chiu et al., 2009; Jain et al., 2009; Wongeakin et al., 2009; Agrawal et al., 2010; Chanpoo et al., 2010;

		Hussein and Abu-Zinadah, 2010; Awasthi et al., 2010; Mahfouz, 2011; Huang et al., 2013; Banafshe et al., 2014; Zhao et al. 2014b
0.5%/day (D.I.) for 2 or 16 weeks/ STZ-induced diabetic rats	Attenuation of osteoclastogenesis	Hie et al., 2009; Chougala et al., 2012
1 g/kg bw/day (p.o.) for 16 weeks/ STZ-induced diabetic rats	Antihyperglycemic, Antioxidant	Gupta et al., 2011
80 mg/kg bw/day (p.o.) for 15 days/ high fat-fed rats	Antihyperglycemic, Antioxidant	El-Moselhy et al., 2011
10 mM/day (i.p.) for 4 weeks/ STZ-induced diabetic mice	Antihyperglycemic, Antioxidant	El-Azab et al., 2011
STZ-induced diabetic rats	Antihyperglycemic, Antioxidant	Acar et al., 2012
60 mg/kg bw/day (p.o.) for 15 days/ STZ-induced diabetic rats	Antihyperglycemic, Ameliorates peripheral diabetic complications	Xavier et al., 2012
30-90 mg/kg bw/day (p.o.) for 31 days/ STZ-induced diabetic rats	Antihyperglycemic, Antihyperlipidemic	Gutierres et al., 2012
80-100 mg/kg bw/day (p.o.) for 8 weeks/ high-fat fed rats or STZ-induced diabetic rats	Antihyperlipidemic, Antioxidant, Ameliorates insulin resistance, Antihyperlipidemic	Hussein and El-Maksoud, 2013; Soetikno et al., 2013
15 mg/5 ml/kg bw/day (p.o.) for 6 weeks/ STZ-induced diabetic rats	Antihyperglycemic, Antioxidant	El-Bahr, 2013
10 mg/kg bw (D.I) for 45 days/ STZ-induced diabetic rats	Antihyperglycemic	Abdel Aziz et al., 2013
60 mg/kg bw/day (p.o.) for 2, 4	Antihyperglycemic,	Kumar et al., 2011; 2013a;

	weeks or 2 months/ STZ-induced diabetic rats	Antioxidant	Abdul-Hamid and Moustafa, 2013; Palma et al., 2014
	100 mg/kg bw/day (p.o.) for 2 weeks/ STZ-induced type 2 diabetic rats	Antihyperglycemic, Ameliorates diabetic neuropathy	
	100 mg/kg bw/day (p.o.) for 3 or 8 weeks/ STZ-induced diabetic rats	Antihyperglycemic, Antioxidant	Rashid and Sil, 2015; Ghosh et al., 2015
Curcumin (photo-irradiated)	10-80 mg/kg bw/day (p.o.) for 45 days/ STZ-induced diabetic rats	Antihyperglycemic, Antioxidant	Mahesh et al., 2004; 2005
Tetrahydrocurcumin	20-80 mg/kg bw/day (p.o.) for 45 days/ STZ-induced diabetic rats or STZ-nicotinamide-induced type 2 diabetic rats	Antihyperglycemic, Improve altered carbohydrate metabolic enzymes, Improves collagen content	Pari and Murugan, 2005; Murugan and Pari, 2006a,b; Murugan et al., 2008; Karthikesan et al., 2010a.b
	300 mg/kg bw/day for 8 weeks/ STZ-induced diabetic rats	Antihyperglycemic, Antihyperlipidemic, Antioxidant	Wongeakin et al., 2009
	100 mg/kg bw/day for 60 days/ high-fat diet-STZ-induced diabetic rats	Antihyperglycemic, Antihyperlipidemic, Antioxidant	Kaur and Meena, 2012
Diosgenin	10 g/kg bw/day for 3 weeks/ STZ-induced diabetic rats	Antihyperglycemic, Antihyperlipidemic	McAnuff et al., 2002; 2006
	0.5%, 2%/day for 4 weeks/ high-fat KK-Ay/Ta Jcl obese diabetic mice	Antihyperglycemic, Insulinotropic	Uemura et al., 2010
	10-60 mg/kg bw/day for 3, 2 weeks or 1, 2 months/ STZ-induced diabetic rats or high-fat diet-STZ-induced diabetic rats	Antihyperglycemic, Antihyperlipidemic, Antioxidant, Ameliorates insulin resistance	Pari et al., 2012; Sangeetha et al., 2013; Saravanan et al. 2014; Tharaheswari et al., 2014; 2015; Naidu et al., 2014

	10 mg/kg bw/day for 1 month/ high-fat diet-STZ-induced diabetic rats	Antihyperglycemic, Antioxidant	Kalailingam et al., 2014
	40 mg/kg bw/day for 45 days or 7 weeks/ (p.o.)/ high-fat diet-STZ-induced diabetic rats	Antihyperglycemic, Antihyperlipidemic, Prevent vascular dysfunction	Roghani-Dehkordi et al., 2014; Hao et al., 2015
	3 mg/kg bw/day for 24 hours/ (p.o.)/ STZ-induced diabetic rats	Antihyperglycemic	Sato et al., 2014
	10-40 mg/kg bw/day for 7 weeks/ (p.o.)/ STZ-induced diabetic rats	Antihyperglycemic, Antihyperlipidemic, Reno-protective, Cardioprotective	Kanchan et al., 2016; Golshahi and Roghani-Dehkordi, 2016
Eugenol	200 mg/kg bw/day for 2 weeks/ (p.o.)/ STZ-induced diabetic rats	Attenuate diabetic neuropathy	Nangle et al, 2006
	80 mg/kg bw/day for 30 days/ (p.o.)/ alloxan-induced diabetic rats	Antihyperglycemic, Antioxidant	Mnafgui et al., 2013
	2.5-10 mg/kg bw/day for 30 days/ (p.o.)/ STZ-induced diabetic rats	Antihyperglycemic, Antioxidant, Improve glycolytic enzyme action	Srinivasan et al., 2014
	20, 40 mg/kg bw/day for 15 weeks/ (p.o.)/ high fat-fed C57BL/6J mice	Antihyperglycemic	Jeong et al., 2014
	10 mg/kg bw/day for 5 days or 6 weeks/ (p.o.)/ STZ-induced diabetic rats	Antihyperglycemic, Antioxidant	Prasad et al., 2015; Singh et al., 2016
Isoeugenol	10 mg/kg bw/day for 2 weeks/ (p.o.)/ STZ-induced diabetic rats	Antioxidant	Rauscher et al., 2001
Galactomannan	2.5, 5% w/w/day/ (p.o.)/ high sucrose-fed rats	Antihyperglycemic, Antihyperlipidemic	Srichamroen et al., 2008
	6 weeks/ (p.o.)/ STZ-induced diabetic	Inhibit maltase, lactase, sucrase	Hamden et al., 2010

	rats	activities	
	500 mg/kg bw/day for 2 hours/ (p.o.)/ alloxan induced diabetic rats	Antihyperglycemic, Antioxidant	Kamble et al., 2013
	250, 500 mg/kg bw/day for 3 weeks/ (p.o.)/ alloxan induced diabetic rats	Antihyperglycemic, Antihyperlipidemic, Antioxidant	Al-Fartosy, 2015
	60, 100 mg/kg bw/day for 12 weeks (p.o.)/ C57BL/6 diabetic mice	Antihyperglycemic, Ameliorate insulin resistance	Kandhare et al., 2015
[6]-Gingerol	60 mg/kg bw/day (i.p.) for 12 days/ db/db mice	Antihyperglycemic, Antihyperlipidemic, Antioxidant	Singh et al. 2009
	75 mg/kg bw /day (p.o.) for 3 weeks/ sodium arsenate hyperglycemic mice	Antihyperglycemic	Chakraborty et al., 2012
	10 μM/ STZ-induced diabetic mice	Antihyperglycemic, Antihypertensive	Namekata et al., 2013
	0.05% (D.I.) for 4 weeks/ db/db mice	Improve glucose utilization	Son et al., 2012
	3 mg/kg bw/day (i.p.) for 8 weeks/ STZ-induced diabetic rats	Antihyperglycemic	Shao et al., 2016
	25, 75 mg/kg bw/ 3 time/week (p.o.) for 16 or 3 weeks/ STZ-induced diabetic rats or C57BL/6J hyperlipidemic mice	Antihyperglycemic, Cardioprotective	El-Bassossy et al., 2016; Sampath et al., 2016; 2017
[6]-Paradol	33.75 mg/kg bw/day (i.p.) for 8 weeks/ high-fat diet-fed mice	Antihyperglycemic, Antihyperlipidemic	Wei et al., 2017
Zingerone	10 mg/kg bw/ day (p.o.) for 4 weeks/ STZ-induced diabetic rats	Antihyperglycemic, Antihyperlipidemic	Jothi et al., 2016a, b
4-Hydroxyisoleucine	50 mg/kg bw/day (i.p.) for 3 hours or 2, 4 weeks/ STZ-induced diabetic rats	Antihyperglycemic, Insulinotropic, Antihyperlipidemic,	Broca et al., 1999; Haeri et al., 2009; Haeri et al., 2012

		Antioxidant	
	20 mg/kg bw/day (i.v.) for 1 hour/ Zucker fa/fa rats	Antihyperglycemic, Insulinotropic	Broca et al., 2004
	50 mg/kg bw/day (p.o.) for 10 days/ high fat-fed hamster or C57BL/KsJ-db/db mice	Antihyperlipidemic	Narender et al., 2006; Singh et al., 2010
	40 mg/kg bw/day (p.o.) for 4 weeks/ alloxan-induced diabetic mice	Antihyperglycemic, Antioxidant	Shah et al., 2009
	50-100 mg/kg bw/day (i.p.) for 3 weeks/ high-fructose diet fed STZ-induced diabetic rats	Antihyperglycemic, Antihyperlipidemic, Ameliorates insulin resistance	Rawat et al., 2014
Piperine	2.5-36 mg/kg bw/day (i.p.) for 2 weeks/ STZ-induced diabetic rats or high-fat diet-fed rats	Antihyperglycemic, Antioxidant, Antihyperlipidemic	Rauscher et al., 2000; Bao et al., 2012; Kharbanda et al., 2016
	40 mg/kg bw/day (p.o.) for 11 weeks/ nicotinamide STZ-induced diabetic rats	Antihyperglycemic, Antihyperlipidemic, Antioxidant	Shah et al., 2010
	25, 50 mg/kg bw/day (p.o) for 4 weeks/ STZ-induced diabetic rats	Antihyperglycemic, Antihyperlipidemic	Kumar et al., 2013b
	20-40 mg/kg bw/day (D.I) for 42 or 45 days/ high-fat diet-fed rats	Antihyperglycemic, Antihyperlipidemic, Antioxidant, Antihypertensive	BrahmaNaidu et al., 2014; Arcaro et al., 2014
	2 mg/kg bw/day (p.o) for 4 weeks/ Alloxan-induced diabetic mice	Antihyperglycemic, Antihyperlipidemic	Boddupalli et al., 2015
Thymoquinone	0.5-6 mg/kg bw/(i.p.)/ non-diabetic rats	Hypoglycemic	Hawsawi et al., 2001
	50 mg/kg bw (p.o.) for 2, 4, 8, 12 weeks or 20 days/ STZ-induced	Antihyperglycemic, Antihyperlipidemic,	Fararh et al., 2005; 2010; Kanter, 2008; 2009;

diabetic hamsters or rats	Decreased hepatic gluconeogenesis, Insulinotropic, Antioxidant	Elmansy and Almasry, 2013
3 mg/kg bw (p.o.) for 30 days/ LETO-STZ-induced diabetic rats	Antihyperglycemic, Insulinotropic, Antioxidant	El-Mahmoudy et al., 2005b
10 mg/kg bw/day (p.o) for 2 weeks/ STZ-induced diabetic rats	Antihyperglycemic, Antioxidant	Hamdy and Taha, 2009
20, 40, 80 mg/kg bw (p.o.) for 45 days/ STZ–nicotinamide-induced diabetic rats	Antihyperglycemic, Insulinotropic, Improved glucose tolerance	Pari and Sankaranarayanan, 2009
3 ml/ kg bw (i.p.) for 6 weeks/ STZ-induced diabetic rats	Antihyperglycemic, Antioxidant, Insulinotropic	Abdelmeguid et al., 2010
20 mg/kg bw (p.o.) for 6 weeks/ STZ-induced diabetic rats	Antihyperglycemic, Improve T cell immune responses	Badr et al., 2011; 2013
80 mg/kg bw (p.o.) for 45 days/ STZ–nicotinamide-induced diabetic rats	Insulinotropic, Antioxidant, Improve plasma glycoprotein metabolism	Sankaranarayanan and Pari, 2011a, b
2.5, 5 mg/kg bw (i.p.) for 5 weeks/ STZ–nicotinamide-induced diabetic rats	Improve spatial memory	Salehi et al., 2012
10, 20 mg/kg bw (p.o.) for 45 days/ STZ–nicotinamide-induced diabetic rats	Antihyperglycemic, Antioxidant	Roghani and Baluchnejadmojarad, 2012

	3 mg/kg bw (p.o.) for 45 days/ STZ–nicotinamide-induced diabetic rats	Insulinotropic, Antioxidant	Hafez, 2013
	5 mg/kg bw (i.p.) for 6 days/ STZ-induced diabetic rats	Antioxidant	Al Wafai, 2013
	20-80 mg/kg bw/day (p.o) for 12 weeks/ STZ-induced diabetic rats	Antihyperglycemic, Ameliorate diabetic cataract, Antioxidant	Fouad and Alwadani, 2015
	60 mg/kg bw/day (p.o) for 2 hours/ STZ-nicotinamide-induced diabetic rats	Antihyperglycemic	El-Ameen et al., 2015
	3 mg/kg bw/day (i.p.) for 56 days/ STZ-induced diabetic rats	Antihyperglycemic, Antioxidant	Sangi et al., 2015
	40 mg/kg bw (p.o.) for 3 weeks/ STZ-induced diabetic rats	Antihyperglycemic, Antihyperlipidemic, Antioxidant	Bashandy et al., 2015
	35 mg/kg bw (i.p.) for 28 days/ STZ-induced diabetic rats	Antihyperglycemic, Antioxidant	Ashour, 2015
Trigonelline	75 mg/kg bw/day (p.o.) for 1 week/ alloxan-induced diabetic rats	Antihyperglycemic, Antioxidant	Shah et al., 2006
	0.056% (D.I.) for 43 days/ Goto-Kakizaki type 2 diabetes rats	Antihyperglycemic, Antihyperlipidemic, Ameliorates insulin resistance, Antioxidant	Yoshinari et al., 2009; Yoshinari and Igarashi, 2010
	10 mg/kg bw/day (p.o.) for 4 weeks/ alloxan-induced diabetic rabbits	Antihyperglycemic, Antihyperlipidemic, Antioxidant	Monago and Nwodo, 2010; Hamadi, 2012; Al-Khateeb et al., 2012; Yoshinari et al., 2013

	40 mg/kg bw/day (D.I.) for 4 or 48 weeks/ high-fat diet-STZ-induced diabetic rats	Antihyperglycemic, Antihyperlipidemic, Antioxidant, Ameliorates insulin resistance	Zhou et al., 2011; Zhou and Zhou, 2012; Tharaheswari et al., 2014; 2015
	50, 100 mg/kg bw/day for 4 weeks/ STZ-induced neonatal or alloxan-induced diabetic rats	Antihyperglycemic, Antihyperlipidemic, Antioxidant, Antihypertensive	Ghule et al., 2012; Hamden et al., 2013a,b
	25-100 mg/kg bw/day (p.o.) for 4 weeks/ nicotinamide STZ-induced diabetic rats	Antihyperglycemic, Antihyperlipidemic, Antioxidant	Kamble and Bodhankar, 2013a,b
	150 mg/kg bw/day (p.o) for 4 weeks/ high-fat-fed STZ-induced diabetic rats	Antihyperglycemic, Antihyperlipidemic, Ameliorates insulin resistance	Subramanian and Prasath, 2014a,b
	50 mg/kg bw/day (p.o.) for 4 or 8 weeks/ nicotinamide STZ-induced diabetic rats	Antihyperglycemic, Cadioprotective, Antioxidant	Kamble and Bodhankar, 2014; Folwarczna et al., 2016
	50, 100 mg/kg bw/day (p.o.) for 2 weeks/ fructose-induced insulin resistance rats	Antihyperglycemic, Antihyperlipidemic, Ameliorates insulin resistance	Ramadan et al., 2016
